# Quantifying in vivo kinematics of the wrist during opposite dart-throwing motion: a 4D CT study

**DOI:** 10.1186/s13018-026-06810-7

**Published:** 2026-03-17

**Authors:** Shijie Jia, Ziyue Xiang, Xinzhe Lu, Xinyao Liu, Qipei Wei, Zhixin Wang, Yaobin Yin, Shanlin Chen

**Affiliations:** 1 Department of Hand Surgery, Beijing Jishuitan Hospital, Capital Medical University, Beijing, China; 2 Beijing Research Institute of Traumatology and Orthopaedics, Beijing, China; 3 Peking University Fourth School of Clinical Medicine, Beijing, China

**Keywords:** Wrist joint, In vivo kinematics, Carpal kinematics, Opposite dart-throwing motion, Four-dimensional computed tomography

## Abstract

**Background:**

The kinematics of the wrist have been widely studied in terms of flexion/extension, radial/ulnar deviation, and the dart-throwing motion (DTM); however, the opposite dart-throwing motion (oDTM)—despite its functional importance in daily activities—remains poorly characterized. This study aimed to quantify in vivo kinematics of the radiocarpal and midcarpal joints and to characterize their motion patterns during oDTM.

**Methods:**

In vivo kinematics of eleven wrists from healthy subjects were assessed using four-dimensional computed tomography (4D CT). A custom-designed device guided wrist motion along a plane oriented 45° in supination from the sagittal plane. Three-dimensional bone models were reconstructed to calculate the Euler angles and translational displacements of the third metacarpal, proximal carpal row, and distal carpal row. Throughout the arc of motion, the contributions of the radiocarpal and midcarpal joints were compared.

**Results:**

The actual oDTM of the subjects was performed within a plane supinated 50.3 ± 7.0° [95% confidence interval (CI): 45.6° to 55.0°] from the sagittal plane, with a motion arc of 59.9 ± 10.3° [95% CI 53.0° to 66.8°]. During ulnar extension, the proximal carpal row underwent extension, supination, and ulnar deviation relative to the radius, while the distal carpal row flexed, pronated, and deviated ulnarly relative to the proximal row. During radial flexion, the proximal carpal row flexed, pronated, and deviated radially, except for the lunate which deviated ulnarly. Relative to the scaphoid, the trapezium, trapezoid, and capitate extended, supinated, and deviated radially. The capitate and hamate flexed, supinated, and deviated radially relative to the lunate and triquetrum, respectively. Translational displacements were generally less than 5 mm. Throughout the oDTM, the radiocarpal joint contributed predominantly to extension/flexion, whereas the midcarpal joint contributed substantially to radial/ulnar deviation.

**Conclusion:**

During the oDTM, the radiocarpal joint exhibited motion consistent with the oDTM plane, while the midcarpal joint—particularly on the radial side—demonstrated motion more characteristic of the classical dart-throwing motion (DTM). Both the radiocarpal and midcarpal joints play integral and complementary roles in facilitating wrist oDTM. However, due to the relatively small sample size (*n* = 11) and the homogeneous cohort, the generalizability of these findings to broader or more diverse populations may be limited.

**Supplementary Information:**

The online version contains supplementary material available at 10.1186/s13018-026-06810-7.

## Background

Historically, wrist kinematics has been extensively studied through conventional clinical movement concepts such as flexion/extension and radial/ulnar deviation, giving rise to several theoretical kinematic models of the wrist [1–4]. The dart‑throwing motion (DTM), characterized by minimal movement of the scaphoid and lunate [5, 6], has garnered considerable clinical interest. Evidence of its frequent involvement in activities of daily living (ADLs)—including hammering a nail, throwing a ball, and drinking from a glass—has been reported [7, 8].

In contrast, the opposite dart‑throwing motion (oDTM)—defined as movement from an extended and ulnar‑deviated position to a flexed and radial‑deviated position, also referred to as the “reversed dart‑throwing” plane—has received limited attention in the carpal kinematics literature [6, 9, 10]. Emerging evidence from studies of ADL suggests that oDTM is actively recruited during bimanual, precision, and modern tool‑use tasks [11–13]. Furthermore, in vivo wrist angle measurements obtained via optical motion‑capture systems have highlighted the functional relevance of ulnar extension in everyday activities [14]. However, to date, no quantitative data exist on the in vivo contributions of individual carpal bones—or the relative roles of the radiocarpal and midcarpal joints—during oDTM.

Given the known biomechanical role of the midcarpal joint in DTM, it has been hypothesized that oDTM occurs predominantly at the radiocarpal joint. The present study aims to test this hypothesis by quantifying in vivo kinematics of the radiocarpal and midcarpal joints using four‑dimensional computed tomography (4D‑CT) and to elucidate the underlying motion characteristics of the wrist during oDTM.

## Methods

### Subjects

The study included healthy volunteers aged 18 to 40 years. Exclusion criteria comprised any history of wrist trauma or surgery, as well as symptomatic wrist conditions (e.g., pain, swelling, or instability). Written informed consent was obtained from all participants prior to enrollment. The study protocol received ethical approval. The sample size was determined based on a previous study [17] and was increased to account for potential data loss due to unsatisfactory image quality.

### Motion guidance apparatus

**Fig. 1:**
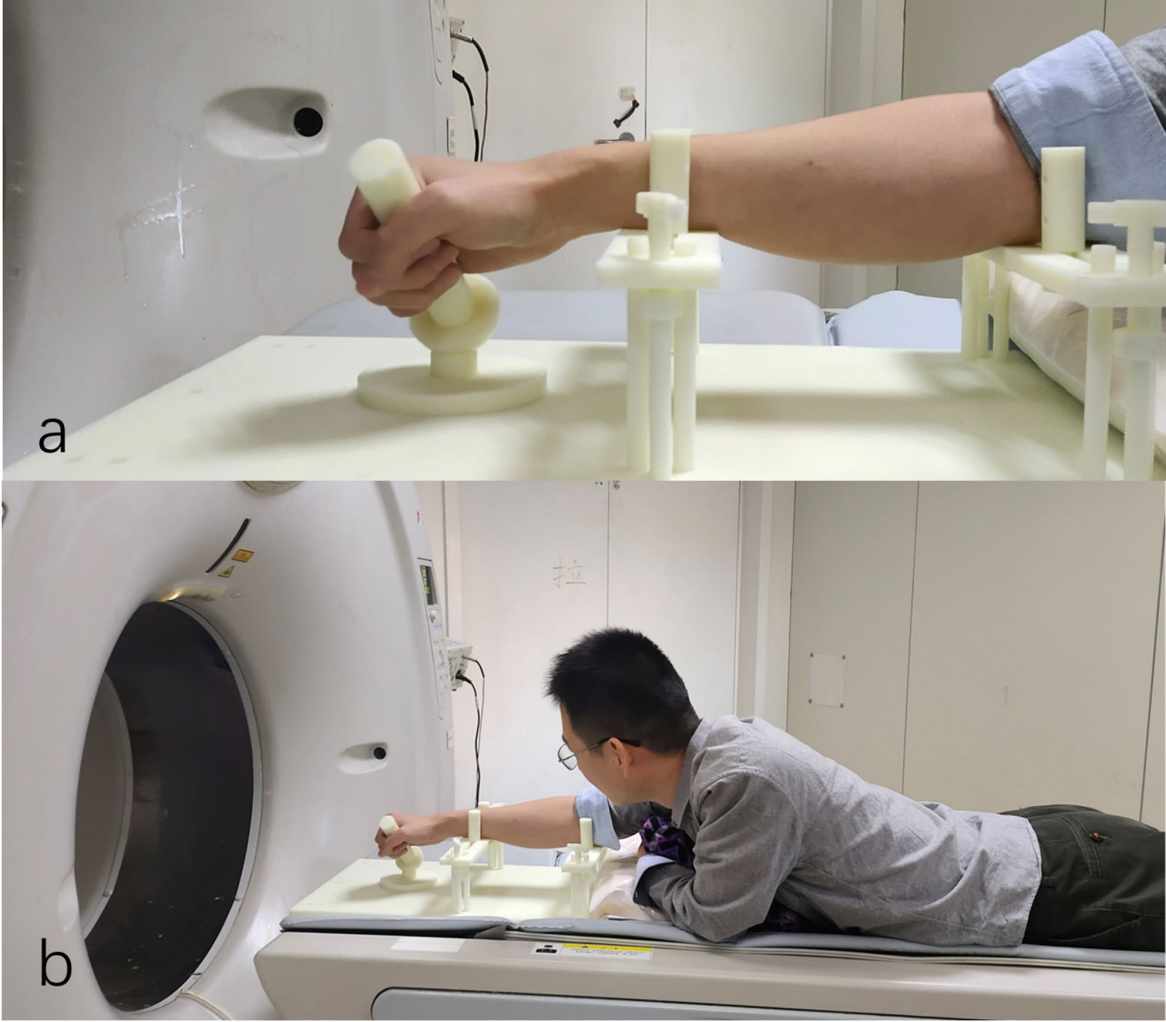
**a** Custom-made motion guidance apparatus used for standardizing hand and wrist kinematics. **b** Representative positioning of a subject during 4D CT acquisition

Inspired by prior methods for quantifying the DTM using a motion‑guiding block [15], a custom device was fabricated to facilitate controlled and reproducible performance of the oDTM (Fig. 1). Participants were instructed to grasp a cylindrical handle (25 mm in diameter, 120 mm in length) in a pronated forearm posture, a configuration known to primarily restrict wrist flexion and ulnar deviation [16]. The handle was fixed at a 45° angle relative to the horizontal plane, thereby constraining wrist motion to a single plane oriented 45° in supination from the sagittal plane. This setup represented the sole restriction on wrist mobility during testing.

To standardize upper‑limb positioning, participants maintained a flexed elbow with the arm oriented within the vertical plane, ensuring consistent forearm pronation throughout the procedure. The neutral wrist position was defined as the posture in which the longitudinal axis of the third metacarpal aligned with that of the radius. Prior to formal data acquisition, each subject completed three practice trials to achieve smooth and continuous motion of the cylinder, analogous to operating a computer mouse. Movement during both practice and actual trials commenced from the operator‑verified neutral wrist position. The motion speed was self-selected by the participants, who were instructed to perform a steady motion, and was not standardized across individuals.

### 4D CT imaging protocol

Imaging was performed on a 320-slice detector CT scanner (Aquilion ONE™, Toshiba Medical Systems, Tokyo, Japan). A 4D CT acquisition protocol was used to capture one complete cycle of the oDTM, defined as movement from an extended, ulnar‑deviated position to a flexed, radial‑deviated position. The dominant wrist of each participant was scanned once. Each scan lasted approximately 5 s and encompassed the region from the distal radius to the third metacarpal. To minimize radiation exposure to non‑target anatomy, lead aprons were placed over adjacent body regions. Following acquisition, three‑dimensional reconstructions were immediately reviewed to verify that the intended wrist motion was adequately captured. The total estimated radiation dose for a single wrist 4D‑CT scan was approximately 0.15 mSv, which falls within an acceptable range and is consistent with previously reported protocols [17, 18]. All imaging data were anonymized prior to storage and subsequent analysis.

### Image processing

Image segmentation and three‑dimensional model reconstruction of the radius, ulna, third metacarpal, and all eight carpal bones were performed using Mimics software (version 21.0, Materialise, Belgium). All bone models were subsequently imported into 3‑Matic software (version 13.0, Materialise, Belgium) for coordinate system construction. A “neutral” reference frame was selected based on the minimal wrist angle observed, defined as the smallest angle between the longitudinal axis of the third metacarpal and the radius. Within 3‑Matic, global registration was applied to align bone models—together with their associated anatomical coordinate systems—across the neutral reference frame and all other motion frames to facilitate kinematic computation.

### Kinematics

**Fig. 2:**
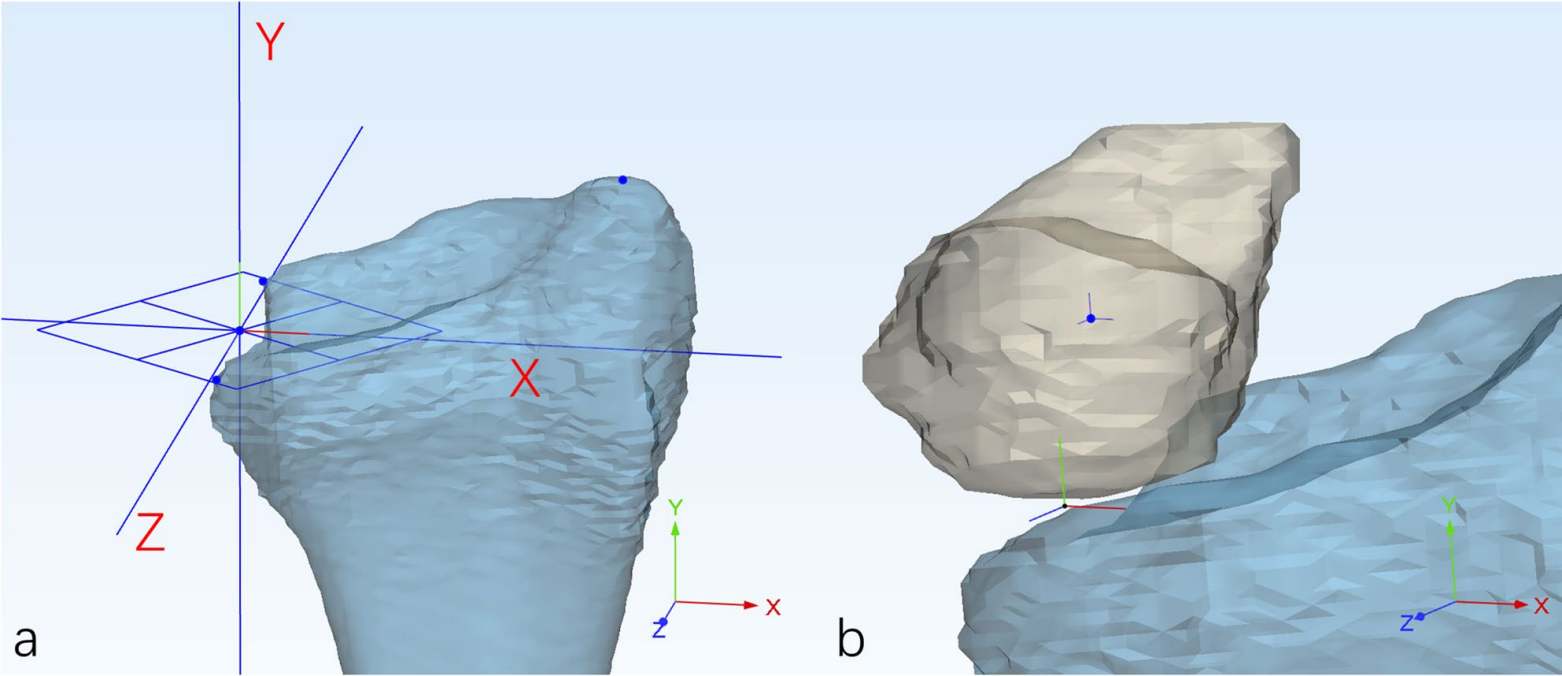
**a** The radius coordinate system was constructed and superimposed onto the global reference frame. **b** The lunate coordinate system was defined parallel to that of the radius

The coordinate system for the distal radius was established according to a previously published 4D CT study [17](Fig. 2). The origin was defined as the midpoint between the volar and dorsal corners of the ulnar side of the radius. A reference plane passing through the origin and perpendicular to the longitudinal axis of the radius was constructed. The Y‑axis was defined perpendicular to this plane, directed distally. The X‑axis passed through the projection of the most distal point of the radial styloid onto the reference plane, oriented radially. The Z‑axis was defined orthogonal to both the X‑ and Y‑axes, directed volarly. Flexion was defined as positive rotation about the X‑axis, extension as negative; ulnar deviation as positive rotation about the Z‑axis, radial deviation as negative; supination as positive rotation about the Y‑axis, and pronation as negative.

For all other bones (carpals and the third metacarpal), a local coordinate system was established with its origin at the centroid of the respective bone, while maintaining axes parallel to those of the radial coordinate system (Fig. 2).

The motion of each bone relative to the radius was decomposed into rotations and translations using an X – Y′ – Z″ Euler angle sequence, with translations defined as displacement of the bone’s center of gravity [19]. To characterize overall wrist kinematics, the helical axis and corresponding rotation angle of the third metacarpal relative to the radius — from neutral to other wrist positions — were calculated. Within this framework, radial flexion and ulnar extension were defined as positive and negative rotations about the helical axis, respectively.

All kinematic calculations were performed using R software (version 4.4.2, R Foundation for Statistical Computing).

Analysis focused on twelve key articulations representative of the functional wrist joint. To characterize the radiocarpal joint, motion of the scaphoid, lunate, and triquetrum relative to the radius (S–R, L–R, T–R) was evaluated. The midcarpal joint was represented by motion of the trapezium, trapezoid, and capitate relative to the scaphoid (Tm–S, Td–S, C–S; collectively referred to as the radial segment), the capitate relative to the lunate (C–L), and the hamate relative to the triquetrum (H–T) (collectively termed the ulnar segment). Relative motion within the proximal carpal row was assessed via the lunate relative to the scaphoid (L–S) and the triquetrum relative to the lunate (T–L). Total wrist motion was defined as the movement of the third metacarpal relative to the radius (M–R) from maximal ulnar extension to maximal radial flexion. The third carpometacarpal joint was represented by the motion of the third metacarpal relative to the capitate (M–C).

Indeed, the clinical interpretation of Euler angles depends on the coordinate system in which they are expressed. For example, while rotation of the capitate about the X‑axis may be described uniformly as “flexion” or “extension,” its kinematic significance differs when defined relative to the lunate (C–L) versus the radius (C–R).

### Statistical analysis

Means, standard deviations, and 95% confidence intervals were calculated for Euler angles and translational displacements across different ranges of wrist motion. A Student’s t-test was applied to compare the designed and the actual planes of wrist motion. To examine the relationship between wrist position and carpal bone kinematics, scatter plots were generated with wrist angle as the independent variable and the rotation/translation of individual carpal bones as the dependent variables. A quadratic polynomial regression model was then fitted to the data for each carpal bone, and the resulting regression curves, along with their corresponding 95% confidence intervals, were plotted to visualize and assess the curvilinear trends.

To compare absolute values of Euler angles and translations between different joints within specific motion ranges, the Friedman rank sum test was employed, followed by post hoc pairwise comparisons using the Wilcoxon signed-rank exact test with Bonferroni correction for multiple comparisons. The threshold of significance was defined as *p* < 0.05. All analyses were conducted using R (version 4.4.2, R Foundation for Statistical Computing).

## Results

Eleven healthy volunteers (6 male, 5 female) aged 23–24 years were enrolled in this study (Table 1). All participants were right‑handed, and the right wrist of each subject was imaged.

The actual motion plane of the oDTM was oriented 50.3 ± 7.0° [95% CI 45.6° to 55.0°] in supination relative to the sagittal plane, which differed significantly from the intended 45° supinated plane (*p* = 0.032). Within this inclined plane, the total angular excursion of the wrist—represented by motion of the third metacarpal—was 59.9 ± 10.3° [95% CI 53.0° to 66.8°]. This motion arc comprised 14.2 ± 11.2° [95% CI 6.7° to 21.7°] of radial flexion and 46.1 ± 7.3° [95% CI 41.2° to 51.0°] of ulnar extension.

**Fig. 3:**
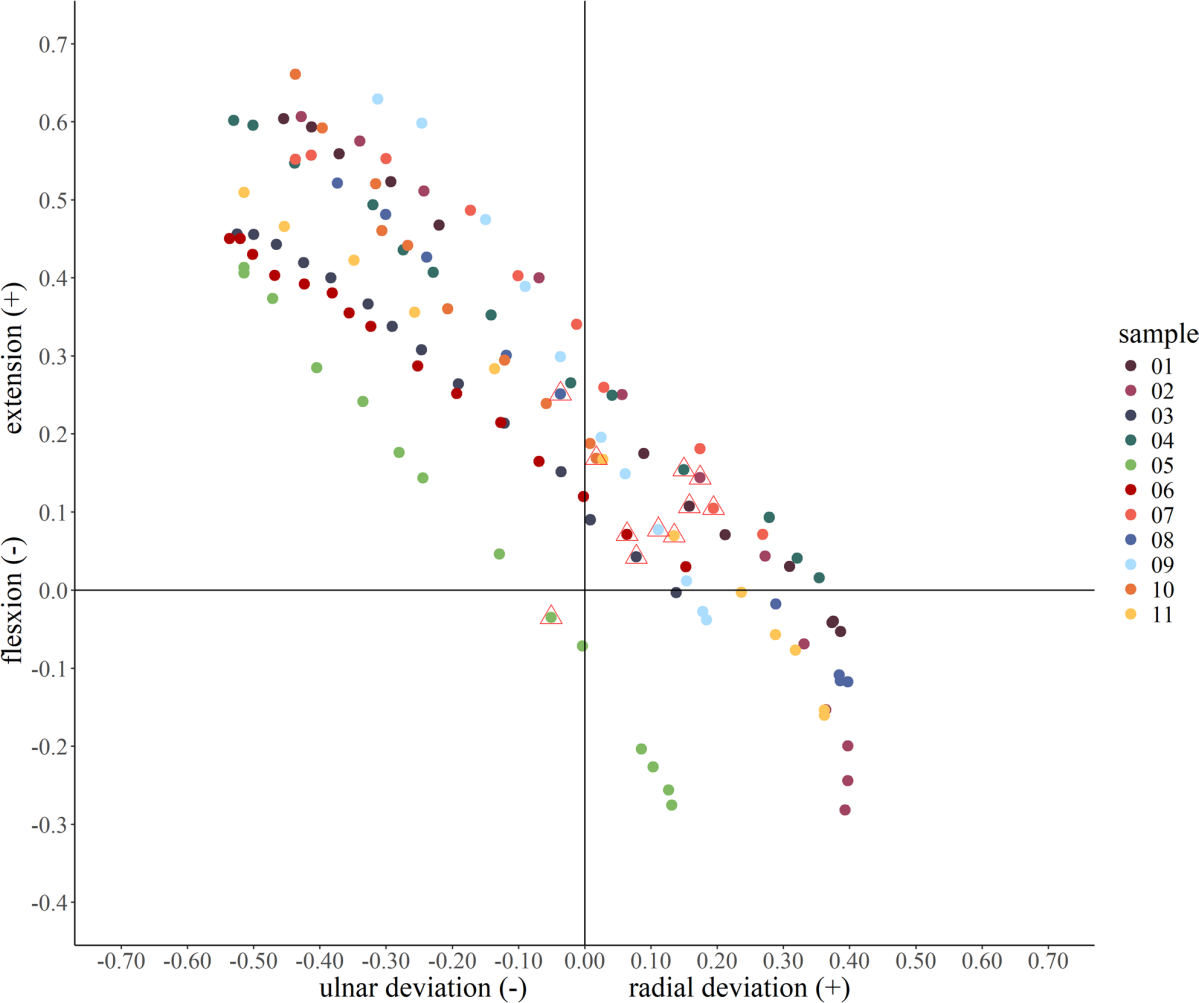
Distal view of the longitudinal axis of the third metacarpal throughout the motion sequence. The neutral frame is indicated by red triangles

Sequential motion frames captured during a representative oDTM cycle are displayed in Fig. 3. The wrist angle measured in the neutral reference frame was 9.5 ± 3.7° [95% CI 7.0° to 12.0°].

**Table 1:** Demographic characteristics of the study participants and wrist motion parameters in the oDTM plane

Participants	gender	age (years)	wrist angle in neutral frame (°)	Motion plane inclination (°)	wrist motion (°)	ulnar extension (°)	radial flexion (°)
01	Male	24	11.0	53.0	69.3	-51.8	17.6
02	Female	24	13.1	42.9	76.2	-48.8	29.0
03	Male	24	5.1	57.2	51.1	-46.5	5.0
04	Female	23	12.4	58.6	71.5	-56.8	14.8
05	Male	24	3.5	44.7	57.7	-40.4	18.1
06	Female	24	5.5	57.7	50.6	-44.9	6.5
07	Female	24	12.8	53.4	52.3	-49.1	5.1
08	Female	24	14.7	51.3	63.2	-28.4	34.9
09	Male	24	7.8	36.7	52.3	-44.5	7.9
10	Male	24	9.8	44.8	46.1	-46.1	-2.5
11	Male	24	8.7	52.8	69.0	-49.9	19.5

The kinematic parameters (Euler angles and translations) for all relevant joints are presented in Figs. 4, 5, 6 and 7 and Supplementary Figs. 1–4.

During wrist ulnar extension, the proximal carpal row exhibited extension, supination, and ulnar deviation. Specifically, the scaphoid, lunate, and triquetrum extended by 32.9 ± 15.9° [95% CI 29.5° to 36.3°], 26.9 ± 14.1° [95% CI 23.9° to 29.9°], and 25.1 ± 13.0° [95% CI 22.3° to 27.8°], respectively, corresponding to 192%, 155%, and 140% of total wrist extension. Translational displacements of the proximal row were generally within 2 mm and approximately 10% of those observed for the third metacarpal, except for a distal translation of the scaphoid (2.6 ± 1.6 mm [95% CI 2.3 to 3.0 mm]) and a proximal translation of the triquetrum (3.2 ± 1.8 mm [95% CI 2.8 to 3.5 mm]), which reached 65% and 63% of the third metacarpal translation, respectively.

In contrast, the distal carpal row flexed, pronated, and deviated ulnarly relative to the proximal row, with translations that were small compared to those of the third metacarpal. Motion between the third metacarpal and the capitate was minimal.

Relative to the scaphoid, the lunate flexed (6.1 ± 5.7° [95% CI 4.9° to 7.3°]), pronated (0.2 ± 1.8° [95% CI −0.2° to 0.6°]), and deviated radially (1.1 ± 3.5° [95% CI 0.3° to 1.8°]), while undergoing ulnar, distal, and dorsal translations of 0.3 ± 0.5 mm [95% CI: 0.2 to 0.4 mm], 0.6 ± 0.8 mm [95% CI 0.4 to 0.7 mm], and 1.3 ± 0.9 mm [95% CI 1.1 to 1.5 mm], respectively.

Relative to the lunate, the triquetrum flexed (1.8 ± 4.6° [95% CI 0.8° to 2.7°]), supinated (0.9 ± 4.0° [95% CI: 0.0° to 1.8°]), and deviated ulnarly (1.9 ± 3.2° [95% CI 1.2° to 2.6°]), accompanied by ulnar, proximal, and volar translations of 0.8 ± 0.5 mm [95% CI 0.7 to 0.9 mm], 0.7 ± 0.5 mm [95% CI 0.6 to 0.8 mm], and 0.4 ± 0.5 mm [95% CI 0.3 to 0.6 mm], respectively.

During wrist radial flexion, the proximal carpal row underwent flexion, pronation, and radial deviation, with the exception of the lunate, which demonstrated ulnar deviation. The scaphoid, lunate, and triquetrum flexed by 14.6 ± 9.2° [95% CI 11.4° to 17.8°], 9.6 ± 6.4° [95% CI 7.4° to 11.9°], and 7.5 ± 6.1° [95% CI 5.4° to 9.6°], respectively, equivalent to 153%, 96%, and 79% of total wrist flexion. Translational motion within the proximal row remained within 2 mm.

Relative to the scaphoid, the trapezium, trapezoid, and capitate extended, supinated, and deviated radially. In contrast, relative to the lunate and triquetrum, the capitate and hamate flexed, supinated, and deviated radially, respectively. Translations of the distal carpal row relative to the proximal row did not exceed 2.2 mm, and motion between the third metacarpal and the capitate was again minimal.

Relative to the scaphoid, the lunate extended (4.9 ± 3.8° [95% CI 3.6° to 6.2°]), supinated (2.1 ± 1.8° [95% CI 1.5° to 2.7°]), and deviated ulnarly (0.4 ± 2.6° [95% CI −0.5° to 1.3°]), accompanied by radial, distal, and volar translations of 0.4 ± 0.3 mm [95% CI 0.3 to 0.5 mm], 0.5 ± 0.6 mm [95% CI 0.3 to 0.7 mm], and 0.8 ± 0.5 mm [95% CI 0.6 to 1.0 mm], respectively.

Relative to the lunate, the triquetrum extended (2.1 ± 2.9° [95% CI 1.1° to 3.1°]), pronated (1.6 ± 2.9° [95% CI 0.60° to 2.6°]), and deviated radially (5.1 ± 3.2° [95% CI 4.0° to 6.2°]), with translations of 0.8 ± 0.5 mm [95% CI 0.7 to 1.0 mm] radially, 0.8 ± 0.5 mm [95% CI 0.7 to 1.0 mm] distally, and 0.0 ± 0.3 mm [95% CI −0.1 to 0.1 mm] dorsally.

**Fig. 4:**
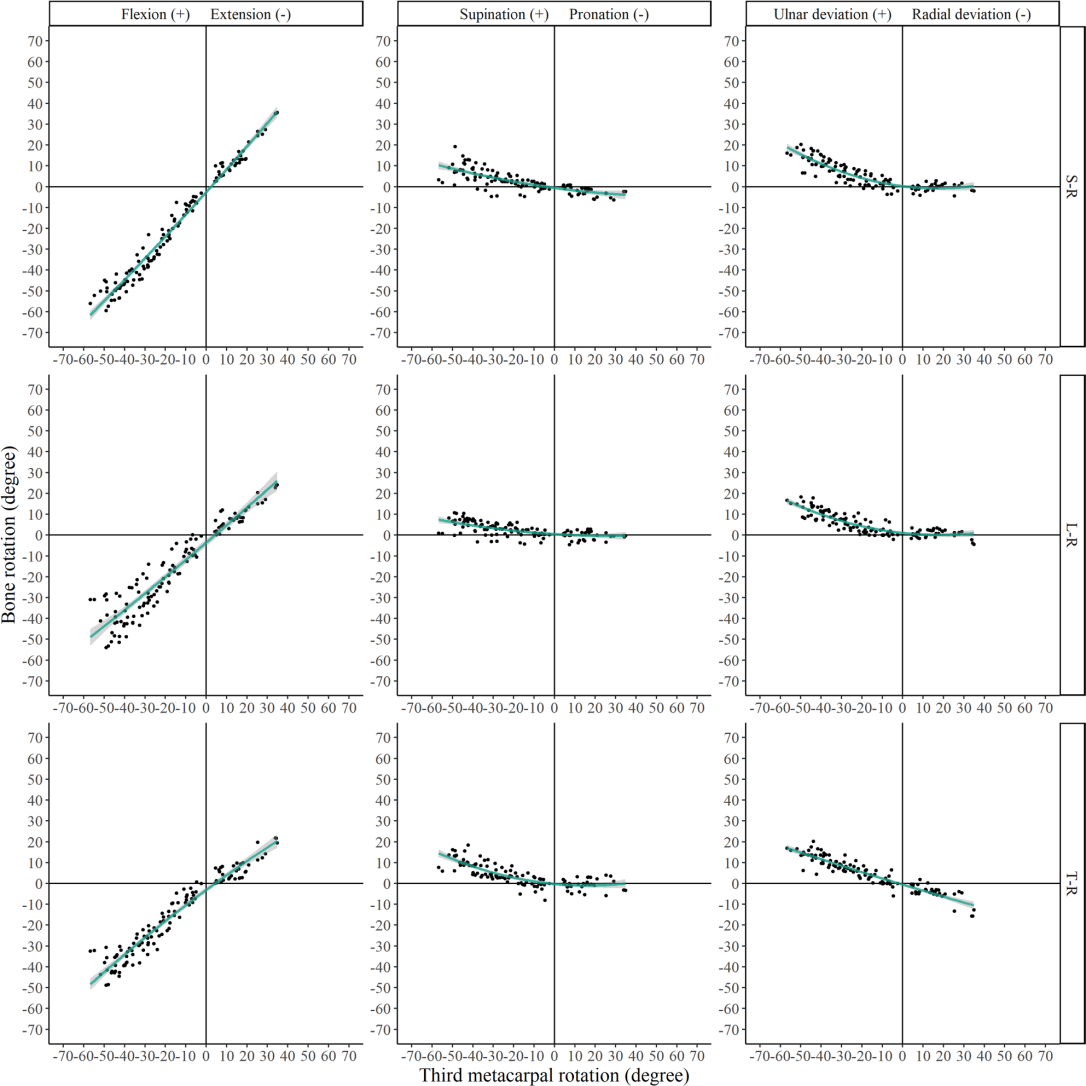
Euler angles of scaphoid, lunate, and triquetrum relative to the radius (denoted as S–R, L–R, and T–R, respectively)

**Fig. 5:**
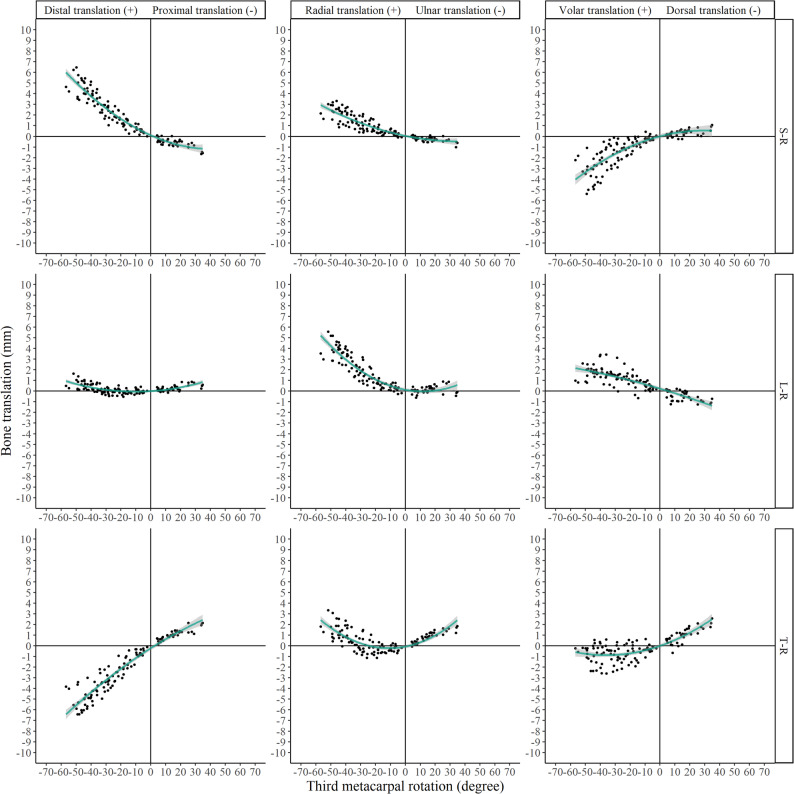
Translations of the scaphoid, lunate, and triquetrum relative to the radius (denoted as S–R, L–R, and T–R, respectively)

**Fig. 6:**
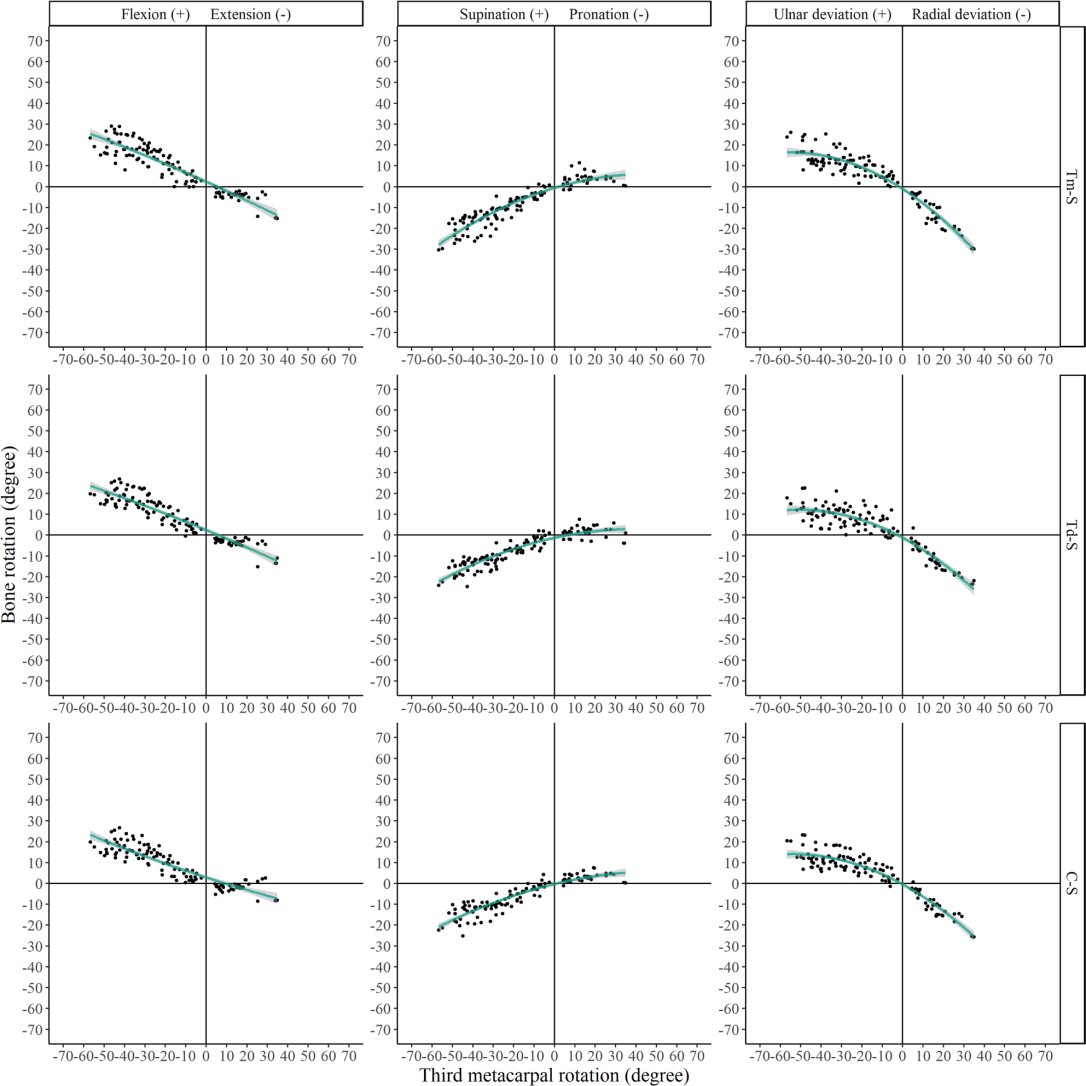
Euler angles of trapezium, trapezoid, and capitate relative to the scaphoid (denoted as Tm-S, Td-S, C-S, respectively)

**Fig. 7:**
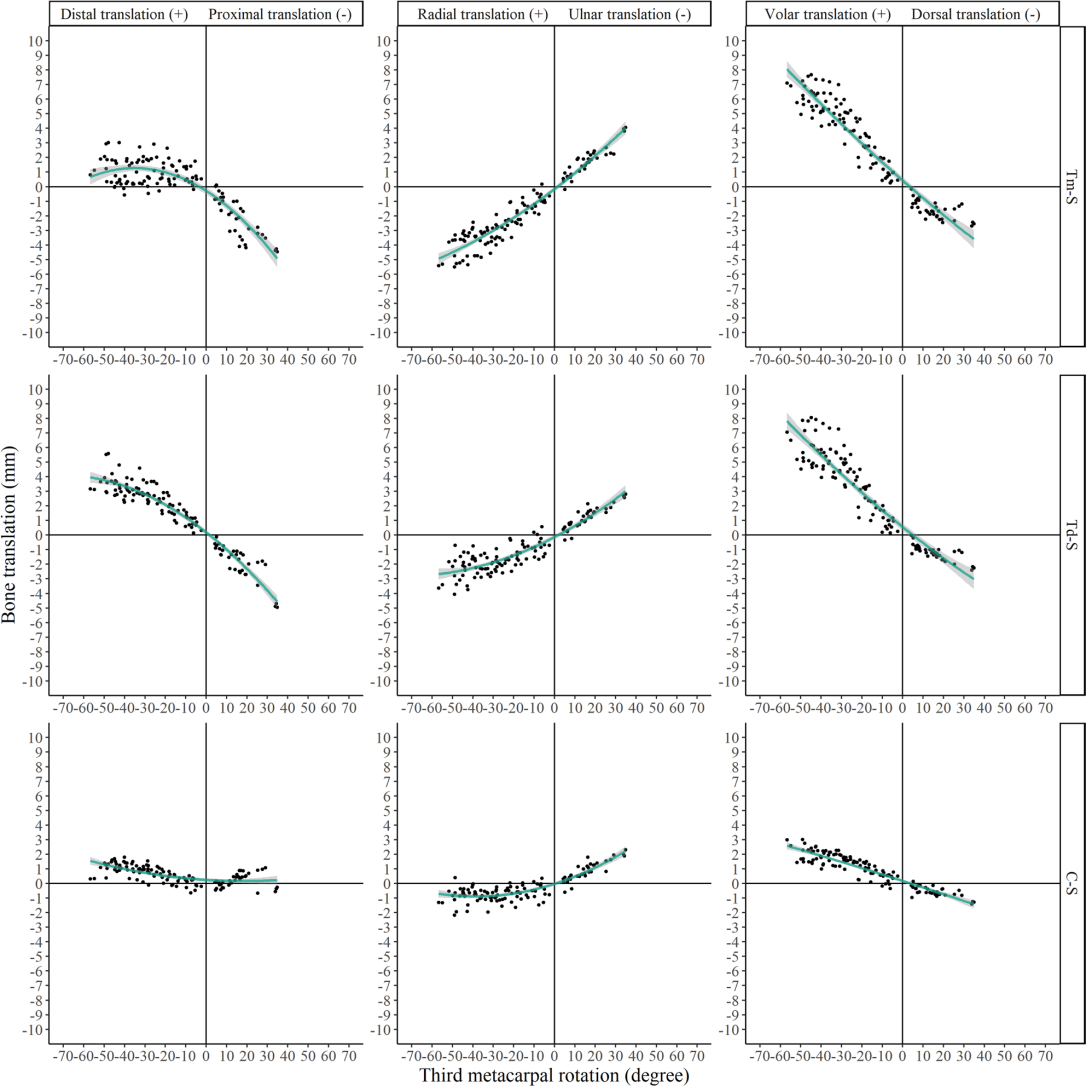
Translations of trapezium, trapezoid, and capitate relative to the scaphoid (denoted as Tm-S, Td-S, C-S, respectively)

**Fig. 8:**
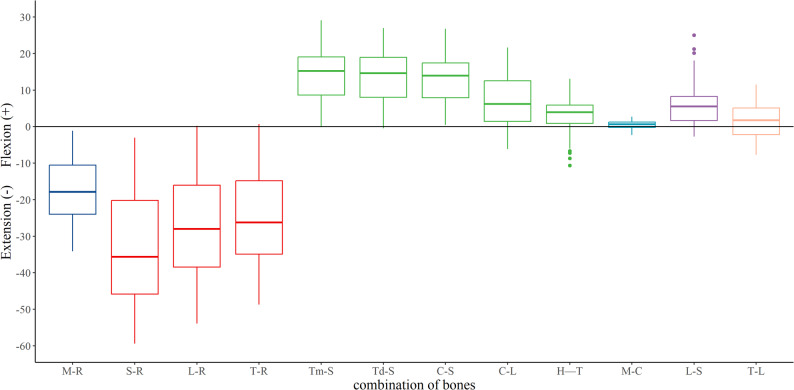
Extension–flexion across the twelve analyzed joints during wrist ulnar extension

**Fig. 9:**
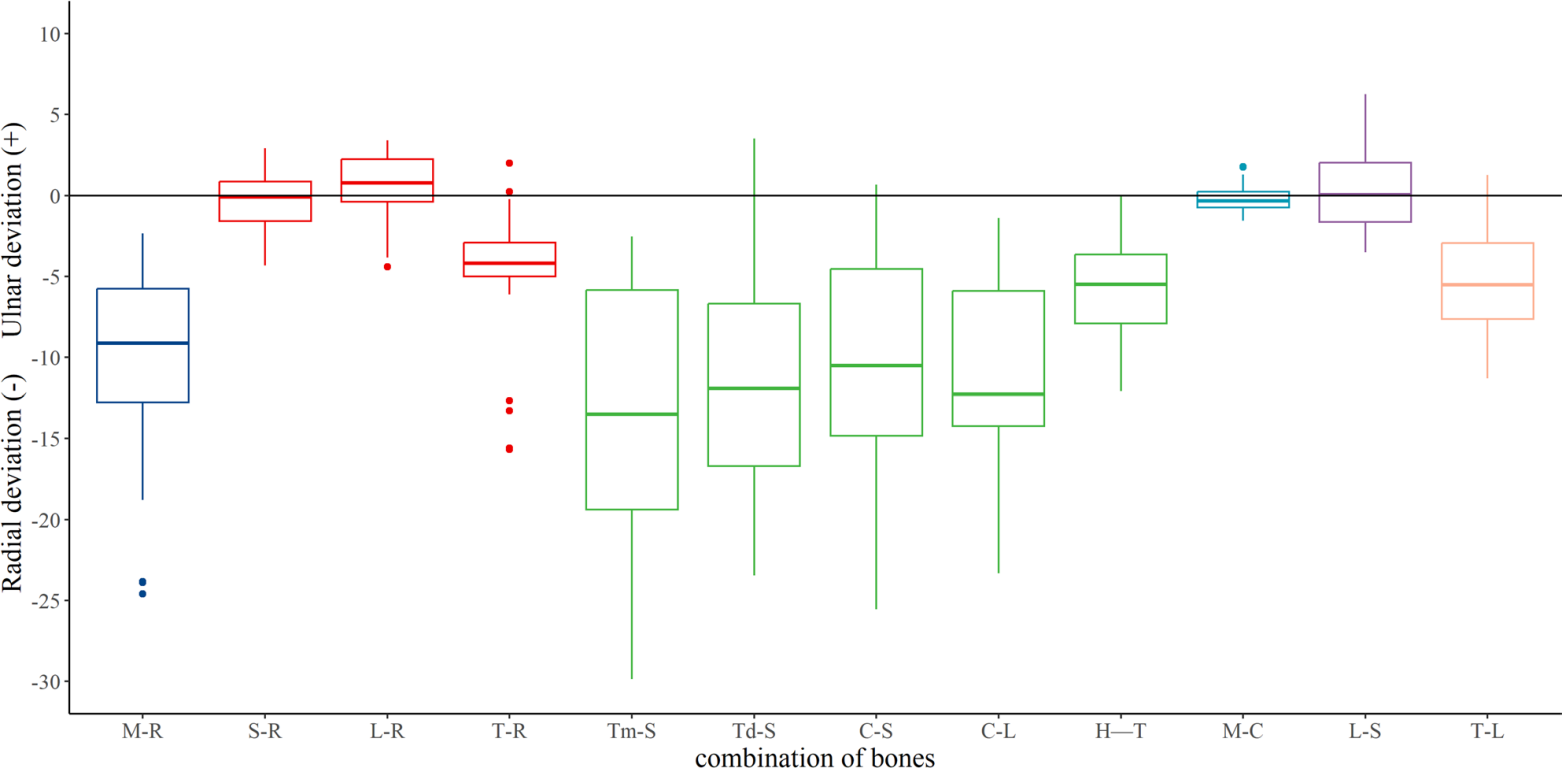
Radial–ulnar deviation across the twelve analyzed joints during wrist radial flexion

During wrist ulnar extension, the radiocarpal joint exhibited significantly greater extension/flexion than the midcarpal joint (Fig. 8) and significantly less supination/pronation. During radial flexion, the radiocarpal joint demonstrated significantly less ulnar/radial deviation (Fig. 9). The radial side of the midcarpal joint showed greater radial/ulnar and volar/dorsal translations than its ulnar side.

Distal/proximal translations of the lunate relative to the radius were significantly smaller than those of the scaphoid and triquetrum. A similar pattern was observed when comparing distal/proximal translations of the capitate relative to the lunate with other articulations within the midcarpal joint. All Friedman rank-sum tests yielded p‑values < 0.05. Additional comparative results and corresponding p‑values are provided in Supplementary Figs. 5–14 and Supplementary Tables 1–5.

When ulnar extension was stratified into subranges (< 30°, > 30°, and extreme ulnar extension) and radial flexion into subranges (< 15°, > 15°, and extreme radial flexion), the kinematic patterns described above remained consistent across all subdivisions.

## Discussion

The present study quantified in vivo carpal kinematics during oDTM using 4D CT. The key findings can be summarized as follows: (1) the radiocarpal joint moved in accordance with the oDTM pattern, exhibiting substantial flexion–extension motion; (2) the midcarpal joint primarily followed the DTM pattern, with pronounced radial–ulnar deviation; (3) within the midcarpal joint, the ulnar segment demonstrated a shift from the DTM toward the oDTM trajectory; and (4) limited motion was observed between individual bones of the proximal carpal row. These findings provide a foundation for understanding the functional roles of individual carpal structures during oDTM, as discussed in the following sections.

### Total wrist motion

In cadaveric 4DCT studies, wrist joint simulators have proven effective in obtaining dynamic image sequences [5, 20–22]. However, standardizing wrist motion in oblique planes during in vivo investigations remains challenging. In the current study, a custom motion‑guidance device was employed to constrain wrist motion to a predefined oblique plane. The actual plane of oDTM was oriented 50.3° from the sagittal plane, a value significantly different from the intended 45° target. This discrepancy is considered acceptable and is likely attributable to the manual establishment of the radial coordinate system. The total range of oDTM measured 59.9 ± 10.3° [95% CI: 53.0° to 66.8°], whereas the DTM has been reported to exceed approximately 100° [15, 23–25]. The relatively constrained arc of oDTM was anticipated. Nevertheless, a motion range of 60° may still hold functional relevance in certain pathological contexts.

For each specimen, a neutral reference frame was selected from the 4D‑CT image series to serve as the kinematic neutral position. Although static CT scanning of the wrist in a neutral posture offers a clear and convenient alternative [26], it was not adopted in this study due to radiation‑safety considerations. Given that the neutral position was operator-calibrated during motion guidance, some deviation from an ideal neutral posture is inevitable, and here an acceptable mean deviation of 9.5 ± 3.7° [95% CI: 7.0° to 12.0°] was observed.

### The role of oDTM in proximal carpal row stability

During DTM, the proximal carpal row is known to remain relatively stable. Our findings demonstrate high mobility of the proximal row during oDTM, characterized by a large amplitude of extension/flexion. This observation aligns with the clinical experience that wrist clunking occurs more evidently during such movements [9]. Furthermore, prior studies have noted similarities in scaphoid flexion and extension patterns between conventional flexion/extension and ulnar‑extension/radial‑flexion motions [6], a finding that is consistent with the kinematic behavior observed in the present study.

The oDTM, or “reverse” dart‑throwing motion, has been proposed to protect against lunotriquetral (LTq) dynamic instabilities by engaging the extensor carpi ulnaris (ECU) and flexor carpi radialis (FCR) [27]. However, this interpretation remains tentative given the documented variability in muscle activation patterns [28]. In the present study, during ulnar extension the lunate primarily flexed relative to the scaphoid, whereas motion between the triquetrum and lunate was minimal. Conversely, during radial flexion the lunate predominantly extended relative to the scaphoid, while the triquetrum deviated radially relative to the lunate. These kinematic patterns suggest that oDTM may indeed help stabilize the LTq joint, yet simultaneously impose greater demand on the scapholunate articulation, potentially exacerbating its dynamic instability. Moreover, the substantial motion observed within the proximal carpal row during oDTM could partially offset any protective effect on the LTq joint.

The percentage contribution of individual joints to overall wrist motion was calculated. For instance, the extension of the scaphoid accounted for 192% of the motion of the third metacarpal relative to the radius. Values exceeding 100% indicate that the individual carpal bone underwent greater angular displacement than the overall wrist motion, reflecting its amplified contribution within the kinematic chain of the wrist. However, caution is warranted when interpreting such percentage-based values; a comprehensive assessment integrating multiple kinematic parameters is necessary. Future studies should consider incorporating more synthesized parameters to facilitate a more intuitive understanding of wrist motion.

### Kinematic comparison between DTM and oDTM

During wrist extension and flexion, the lunate and scaphoid moved in the same direction, with the scaphoid exhibiting greater amplitude; during radial or ulnar deviation, both bones also moved concordantly, though with smaller magnitude and coupled flexion or extension [26]. During DTM, the scaphoid followed the DTM path with reduced excursion, while the lunate exhibited limited motion during ulnar flexion and minimal motion during radial extension [21]. During oDTM, the entire proximal carpal row moved as oDTM relative to the radius, with the scaphoid again displaying greater extension/flexion. During ulnar extension, the midcarpal joint underwent ulnar flexion—consistent with DTM. In radial flexion, the radial segment extended radially, also conforming to DTM, whereas the ulnar segment flexed radially.

Our findings further support the view that the scaphotrapeziotrapezoid and scaphocapitate joints function essentially as a single‑degree‑of‑freedom mechanism aligned with DTM, while the articulations between the distal carpal row and the lunate and triquetrum act as weaker kinematic constraints [29]. For DTM (including DTM‑inclined extension/flexion), the midcarpal joint appears sufficient, requiring minimal contribution from the radiocarpal joint. Conversely, during oDTM (including oDTM‑inclined extension/flexion), the midcarpal joint—constrained primarily by its radial aspect—remains limited to DTM‑like motion, thereby necessitating greater involvement of the radiocarpal joint to achieve the overall oDTM trajectory. Thus, combined radial/ulnar deviation of the wrist appears to result from the integrated contributions of DTM‑dominant motion at the midcarpal joint and oDTM‑dominant motion at the radiocarpal joint.

### Kinematic comparison between radiocarpal and midcarpal joints

The present study enabled a detailed kinematic comparison between the radiocarpal and midcarpal joints during oDTM. In studies of opposite-dart-throwing-motion-inclined flexion–extension motion, the capitate has been reported to remain relatively stationary, with the scaphotrapeziotrapezoid joint appearing “locked” and exhibiting no consistent kinematic pattern; in contrast, the lunate and triquetrum demonstrated aligned motion [29]. In the present study, motion within the midcarpal joint was clearly detectable and, at times, particularly pronounced on the radial side. The articulations involving the trapezium, trapezoid, capitate, and scaphoid appear to contribute substantially to oDTM. Furthermore, during wrist radial flexion, a distinct kinematic difference was observed between the radial and ulnar sides of the midcarpal joint. Our results demonstrate that the radiocarpal joint contributes predominantly to extension/flexion, whereas the midcarpal joint is primarily responsible for ulnar/radial deviation. Notably, motion amplitude tended to be greater on the radial side of the wrist.

These findings argue against a simplified model in which the midcarpal joint exclusively governs DTM and the radiocarpal joint solely governs oDTM. Rather, the wrist functions as an integrated three‑dimensional structure comprising both the radiocarpal and midcarpal joints, emphasizing the concept of intercalated segment [30].

In summary, the midcarpal joint primarily facilitates wrist DTM, while the radiocarpal joint serves as a versatile adaptor, accommodating the remaining motion required for functional tasks.

Clinical relevance.

Substantial extension–flexion motion was observed between the scaphoid and lunate during the oDTM, suggesting that this motion should be minimized in the conservative management of scapholunate (SL) injuries or following SL reconstructive surgeries. In the less common setting of dynamic LTq instability, the potential therapeutic benefit of oDTM may be compromised by the considerable motion of the proximal carpal row relative to the radius.

The wrist clunking observed during oDTM warrants attention in both clinical examination and kinematic analysis. Given its capacity to induce substantial motion of the proximal carpal row across a relatively large arc, the oDTM may offer additional diagnostic insights and enhance clinical understanding of wrist biomechanics.

While preservation of midcarpal joint function enables the frequently utilized DTM, this constrained motion also represents a functional limitation. In contrast, the versatility of the radiocarpal joint may inspire novel strategies for functional preservation. Furthermore, the oDTM trajectory could inform the design of future joint prostheses, offering an additional option for patients and clinicians.

Nevertheless, given the limited understanding of oDTM to date, further clinical observations and well-designed studies are needed to support its translation into clinical practice.

4D CT remains a complementary tool rather than a routine part of clinical practice. Its limited adoption stems from the considerable time required for both acquisition and analysis, which constrains its practical clinical effectiveness. Furthermore, identifying the specific pathological motion to target with this modality is often less clear than in a controlled study design. Advancing our understanding of wrist kinetics in specific pathological contexts may help establish clearer indications for its future use.

Limitations.

Several limitations of this study should be acknowledged. First, although the results are considered acceptable, the variability of in vivo motion planes was evident. Second, the lack of a static neutral-position scan may have influenced the calculation of Euler angles and translational displacements. As a result, the oDTM trajectory for each wrist was divided into two phases. Because the phase boundary is sensitive to the definition of neutral position, different definitions could introduce bias into phase-specific comparisons. However, given that our defined neutral position deviated from a true neutral position by only 9.5°, we expect the potential bias to the observed kinematic patterns and relative motion trajectories to be minimal. Third, while the sample size is comparable to that of previous kinematic studies, it remains relatively small and may not fully capture the spectrum of physiological wrist motion. The absence of a post-hoc power analysis to verify sufficient statistical power for detecting differences between joint contributions further warrants caution in result interpretation. Moreover, the homogeneous cohort (all young, right-handed individuals) limits the generalizability of our conclusions to other populations. Fourth, the oDTM was performed with the forearm in a pronated position, which may influence radioulnar ligament length and ulnar variance [31, 32]. Accordingly, the kinematic values reported here may not be directly generalizable to oDTM performed with the forearm in neutral or supinated positions. Finally, due to methodological constraints, the present analysis did not incorporate carpal bone morphology, which could further refine the interpretation of observed kinematics.

## Conclusion

This in vivo 4D CT study delineates the distinct kinematic roles of the radiocarpal and midcarpal joints during the oDTM. The radiocarpal joint predominantly provides extension–flexion, whereas the midcarpal joint facilitates radial–ulnar deviation, with more pronounced motion on the radial aspect. These results underscore the synergistic interaction between both joints in enabling functional wrist mobility and affirm the value of regional kinematic analysis in understanding carpal mechanics.

## Supplementary Information

Below is the link to the electronic supplementary material.

Supplementary Material 1.

## Data Availability

The datasets generated and/or analyzed during the current study are available from the corresponding author on reasonable request.

## Abbreviations

DTM: Dart-throwing motion
oDTM: Opposite dart-throwing motion
4D CT: Four-dimensional computed tomography
CI: Confidence interval
ADLs: Activities of daily living
LTq: Lunotriquetral
ECU: Extensor carpi ulnaris
FCR: Flexor carpi radialis
