# Supplementary material for: Quantifying in vivo kinematics of the wrist during opposite dart-throwing motion: a 4D CT study
Source: J Orthop Surg Res. 2026 Mar 17;21:277. doi: 10.1186/s13018-026-06810-7 (PMC13112708; doi:10.1186/s13018-026-06810-7)
Supplement: Supplementary file 1 — Supplementary Material 1. [file 13018_2026_6810_MOESM1_ESM.docx]

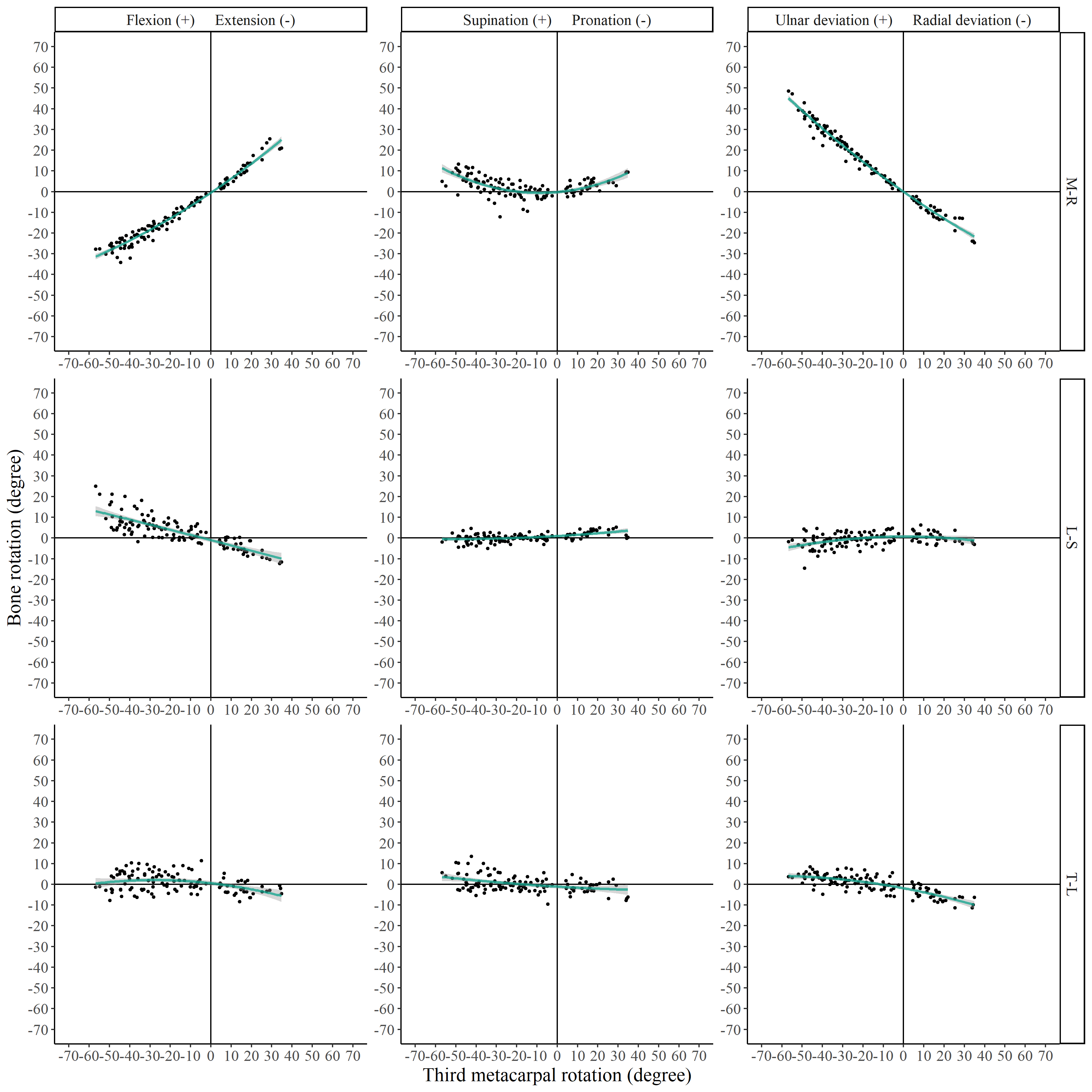


Figure 1. Euler angles of third metacarpal relative to the radius (M-R), lunate relative to scaphoid (L-S) and triquetrum relative to lunate (T-L).


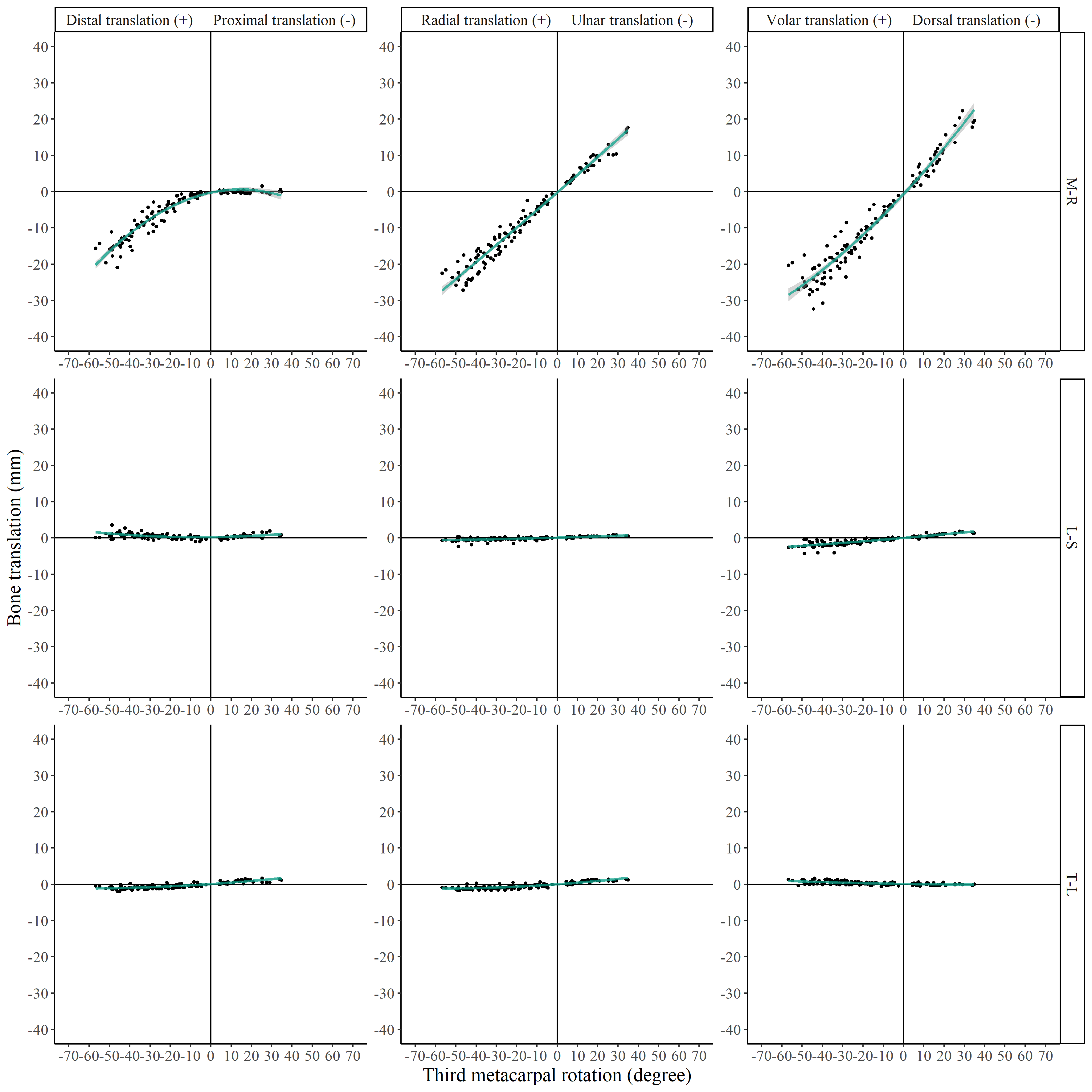


Figure 2. translations of third metacarpal relative to the radius (M-R), lunate relative to scaphoid (L-S) and triquetrum relative to lunate (T-L).


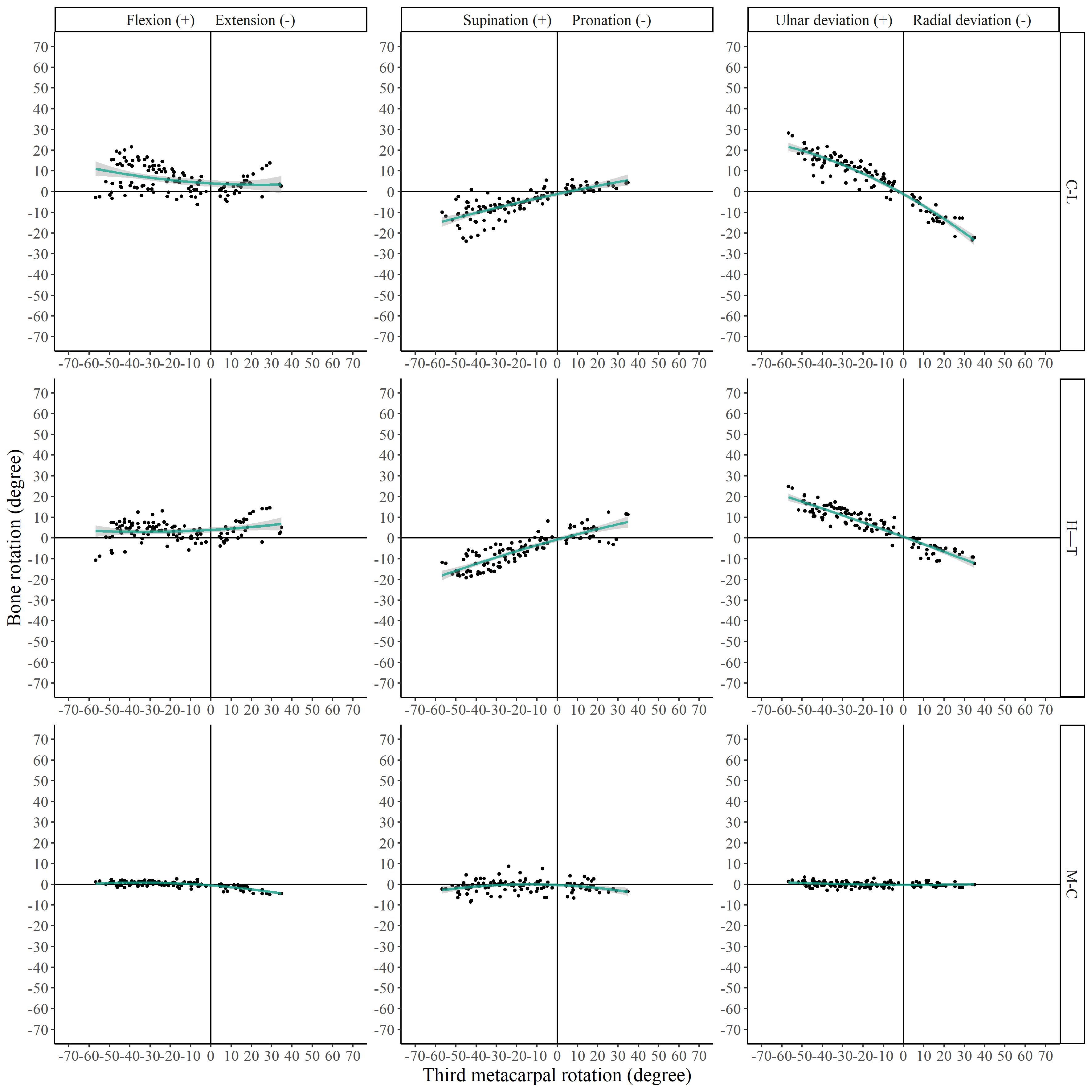


Figure 3. Euler angles of capitate relative to the lunate (C-L), hamate relative to the triquetrum (H-T), third metacarpal relative to the capitate (M-C).


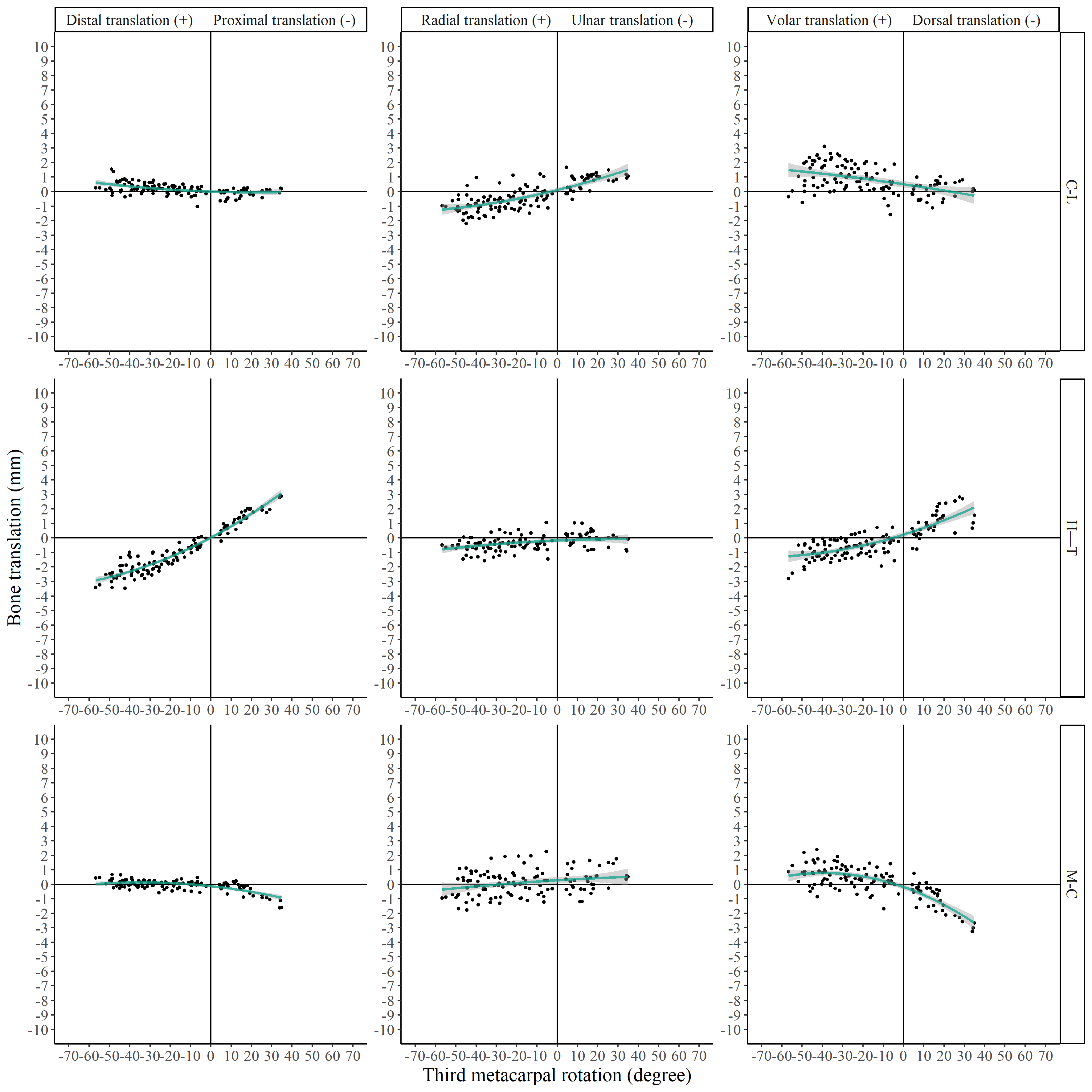


Figure 4. Translations of capitate relative to the lunate (C-L), hamate relative to the triquetrum (H-T), third metacarpal relative to the capitate (M-C).


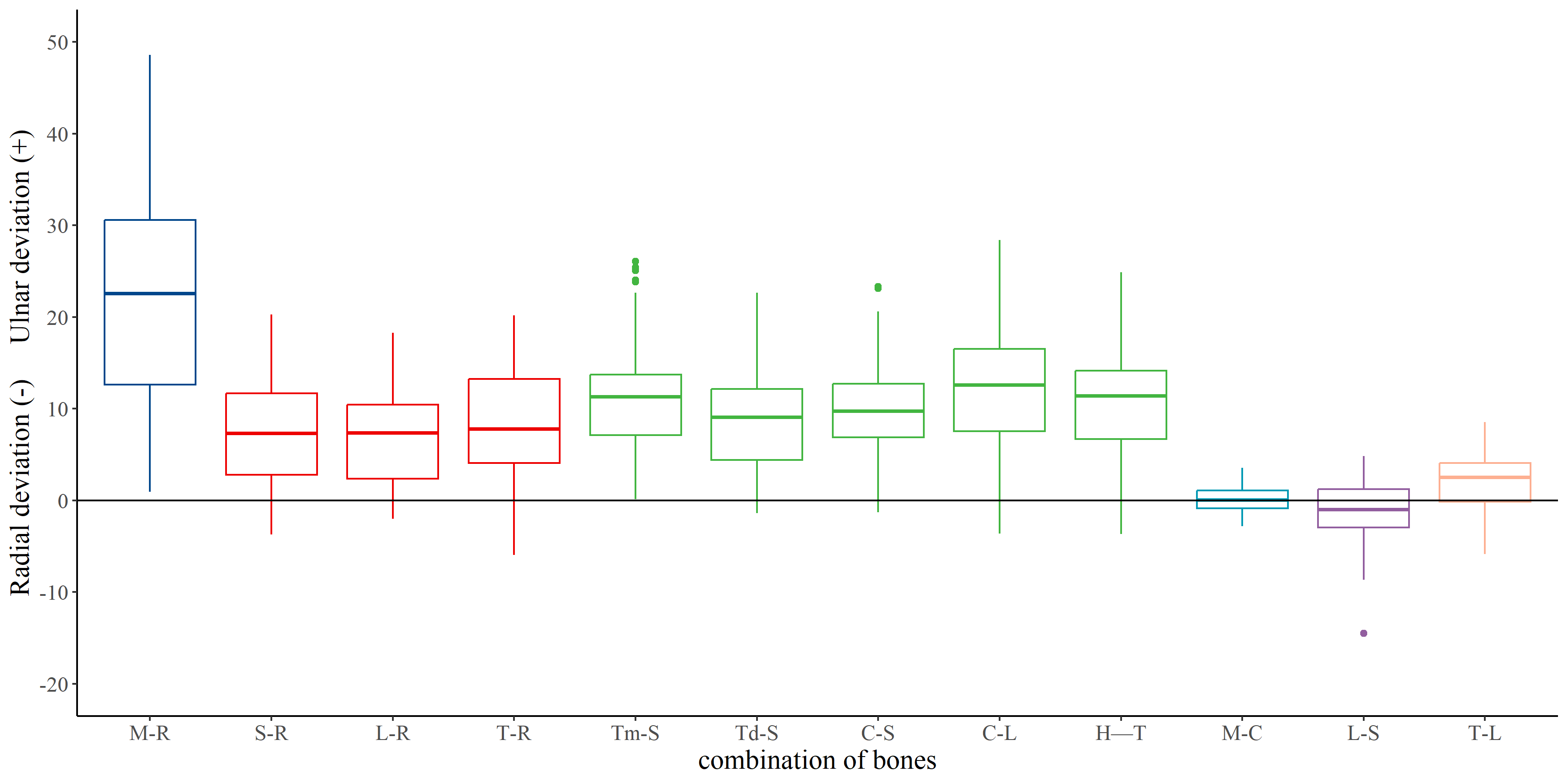


Figure 5. Radial/ulnar deviation of the twelve analyzed joints during wrist ulnar extension.


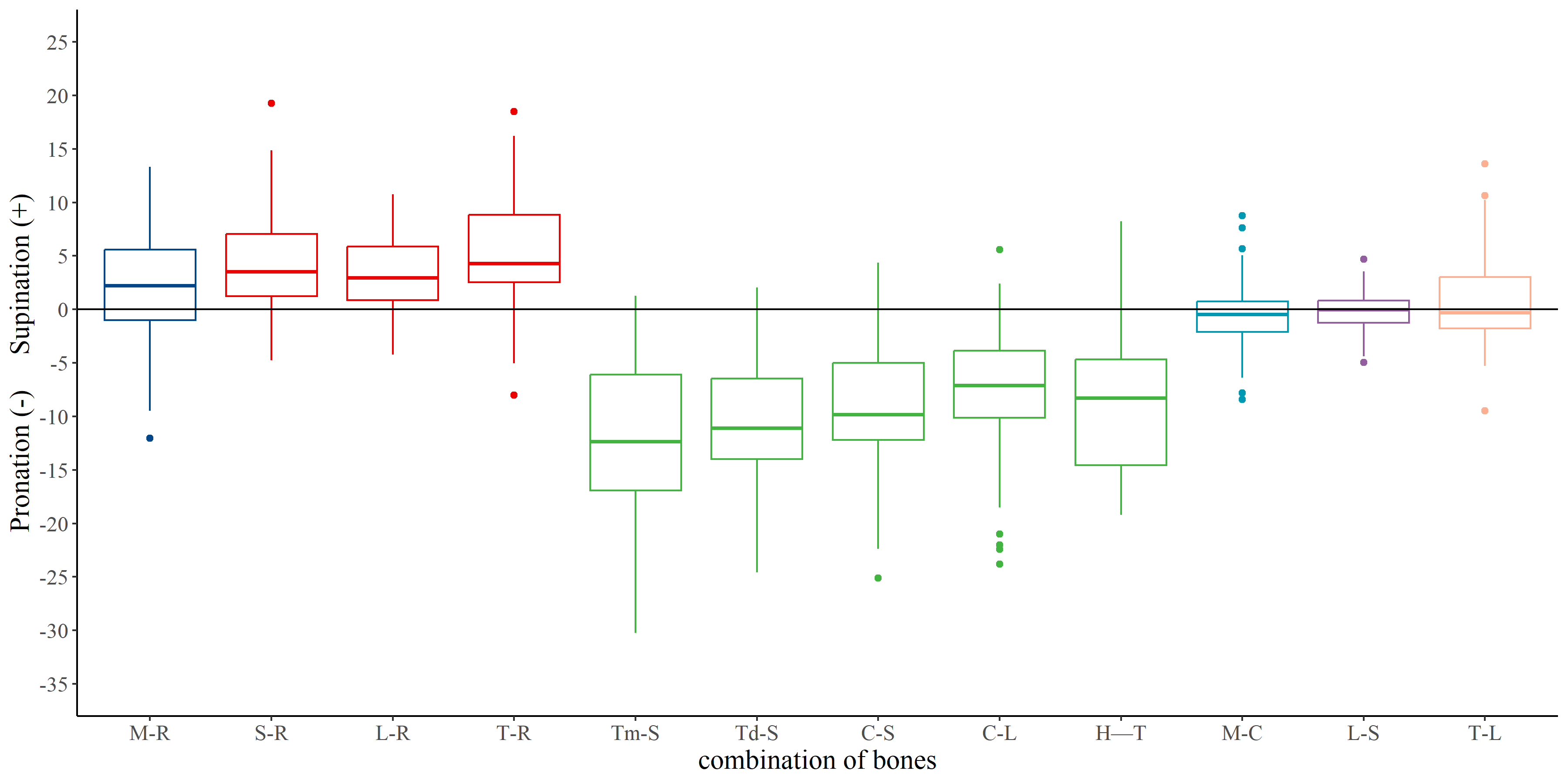


Figure 6. Supination/pronation of the twelve analyzed joints during wrist ulnar extension.


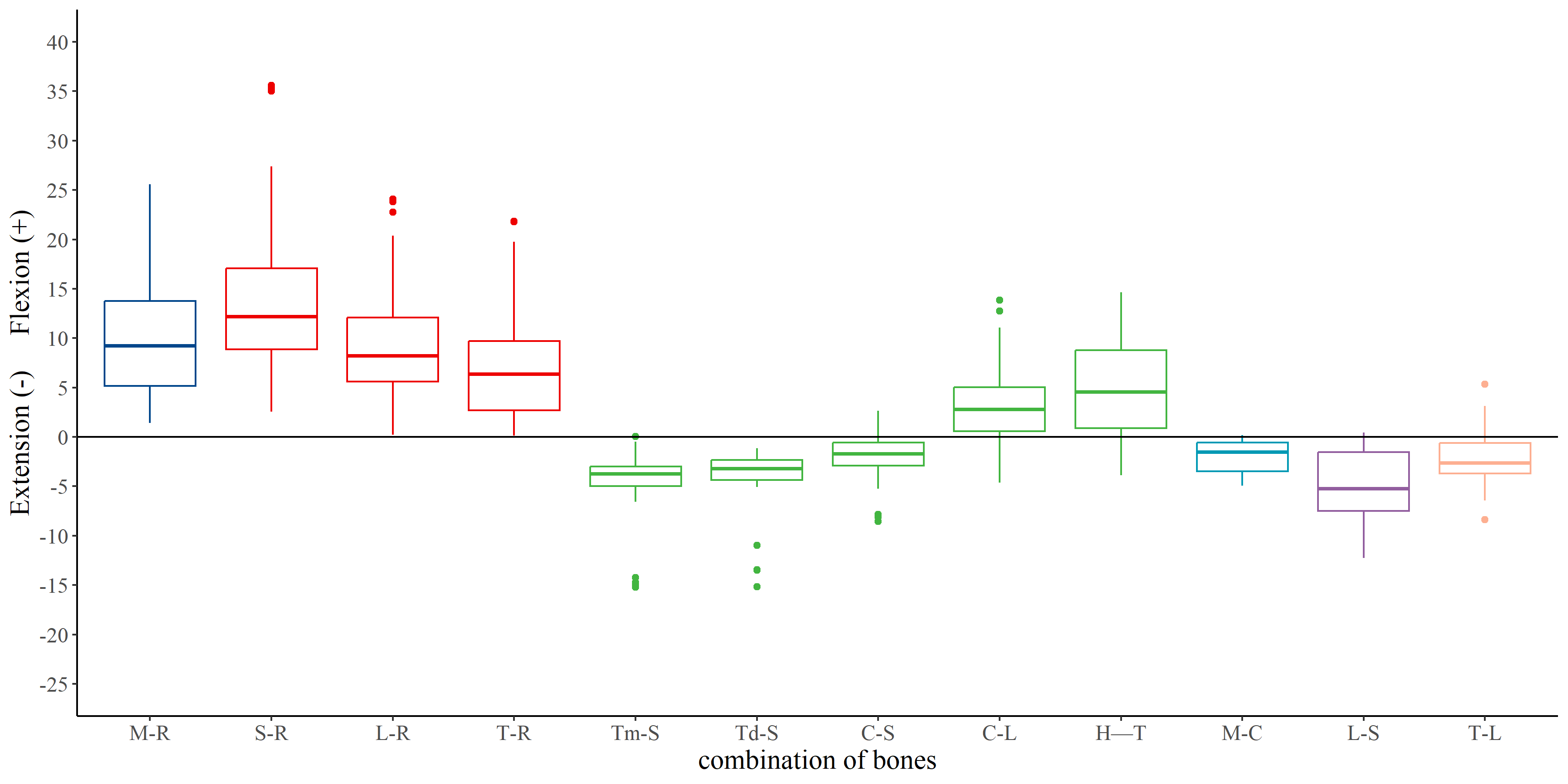


Figure 7. Extension/flexion of the twelve analyzed joints during wrist radial flexion.


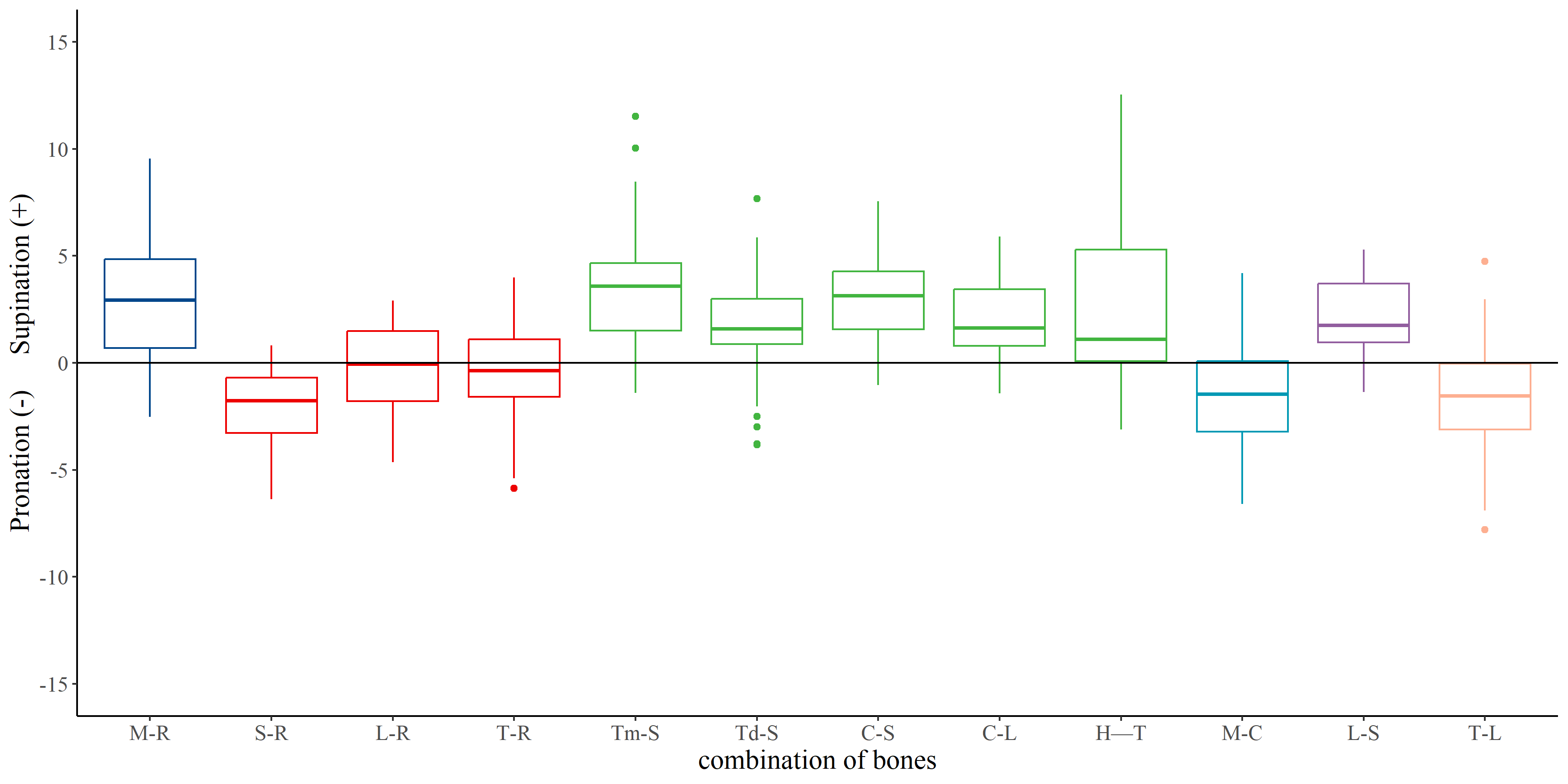


Figure 8. Supination/pronation of the twelve analyzed joints during wrist radial flexion.


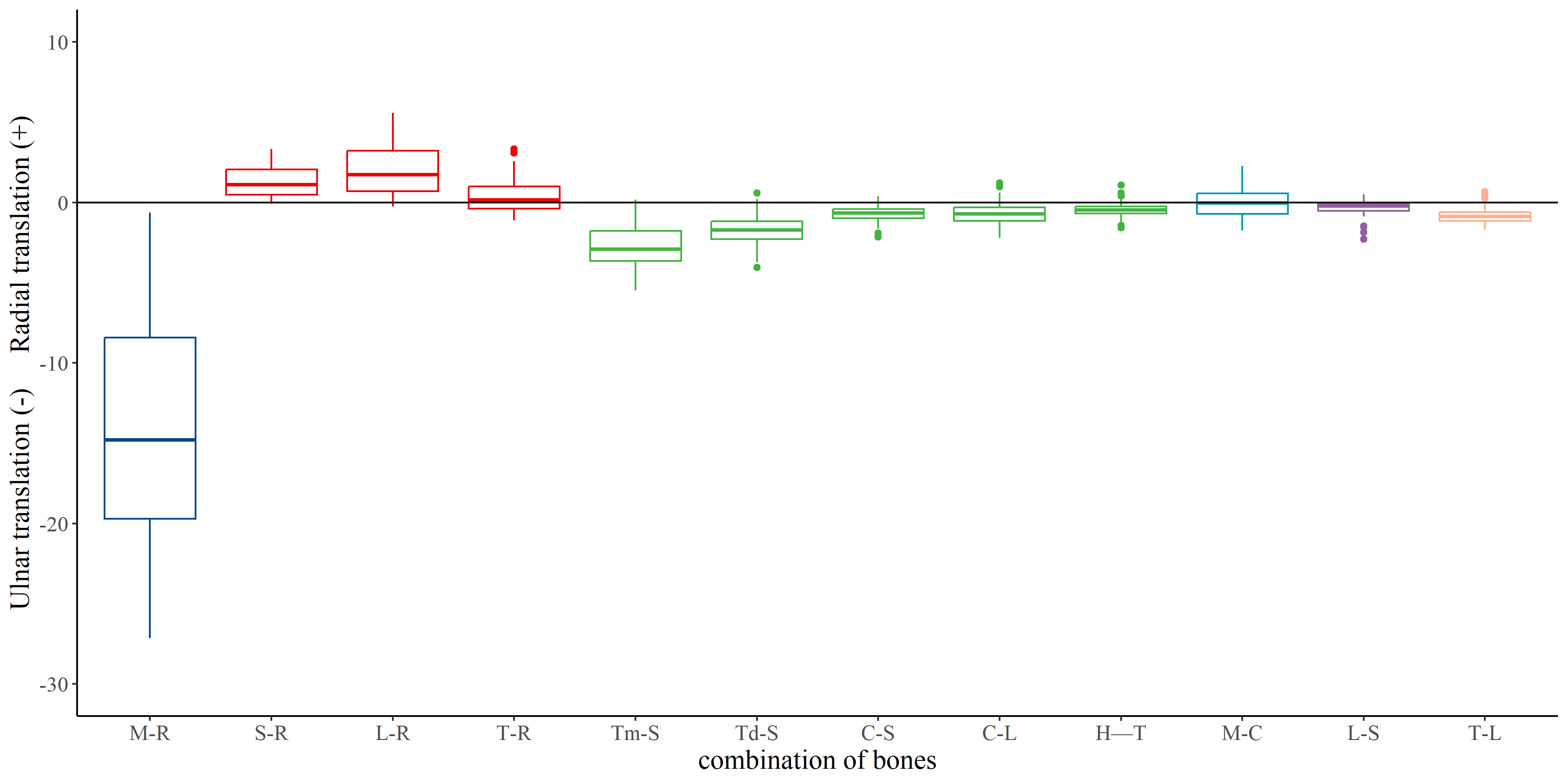


Figure 9. Radial/ulnar translations of the twelve analyzed joints during wrist ulnar extension.


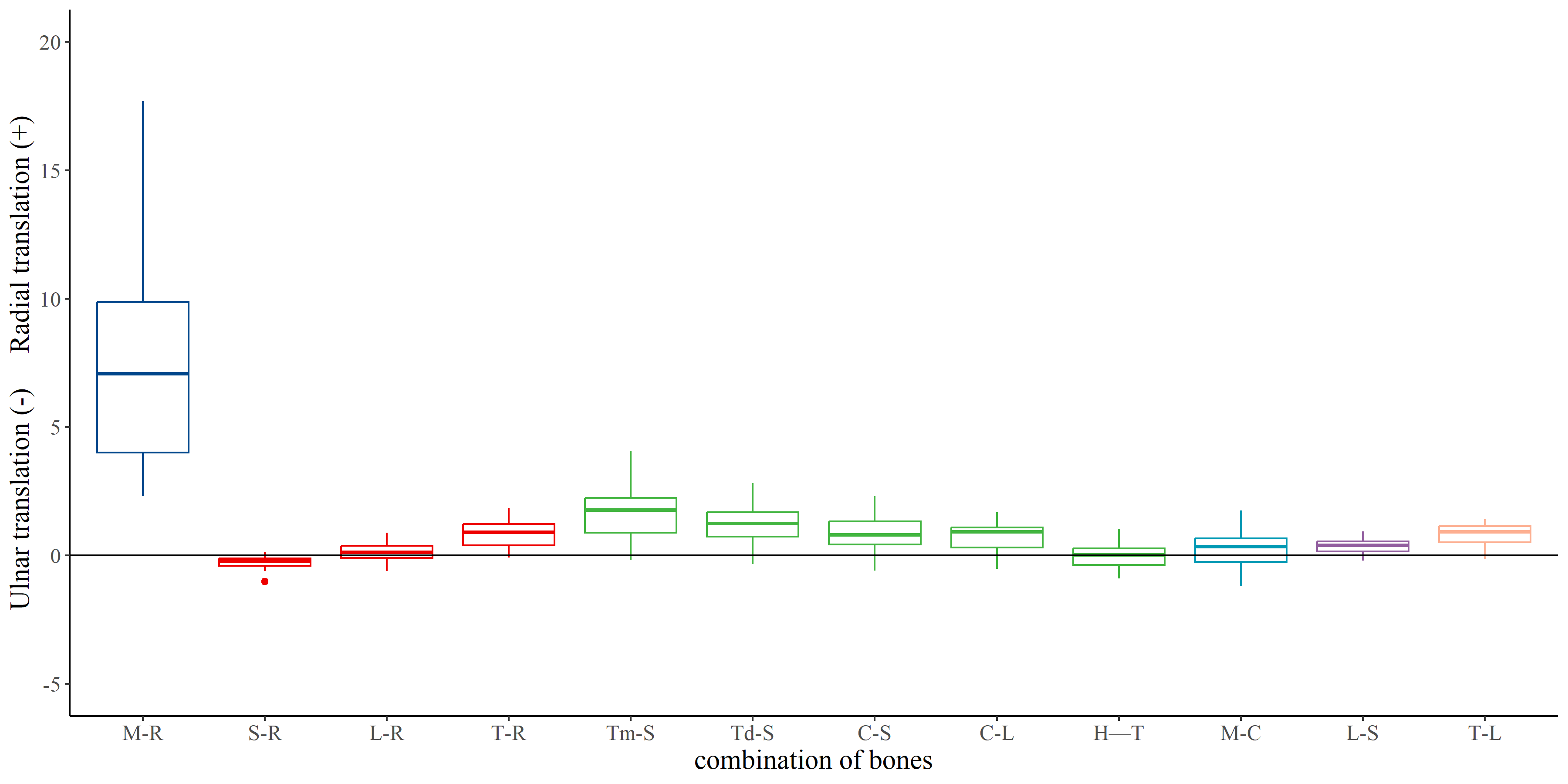


Figure 10. Radial/ulnar translations of the twelve analyzed joints during wrist radial flexion.


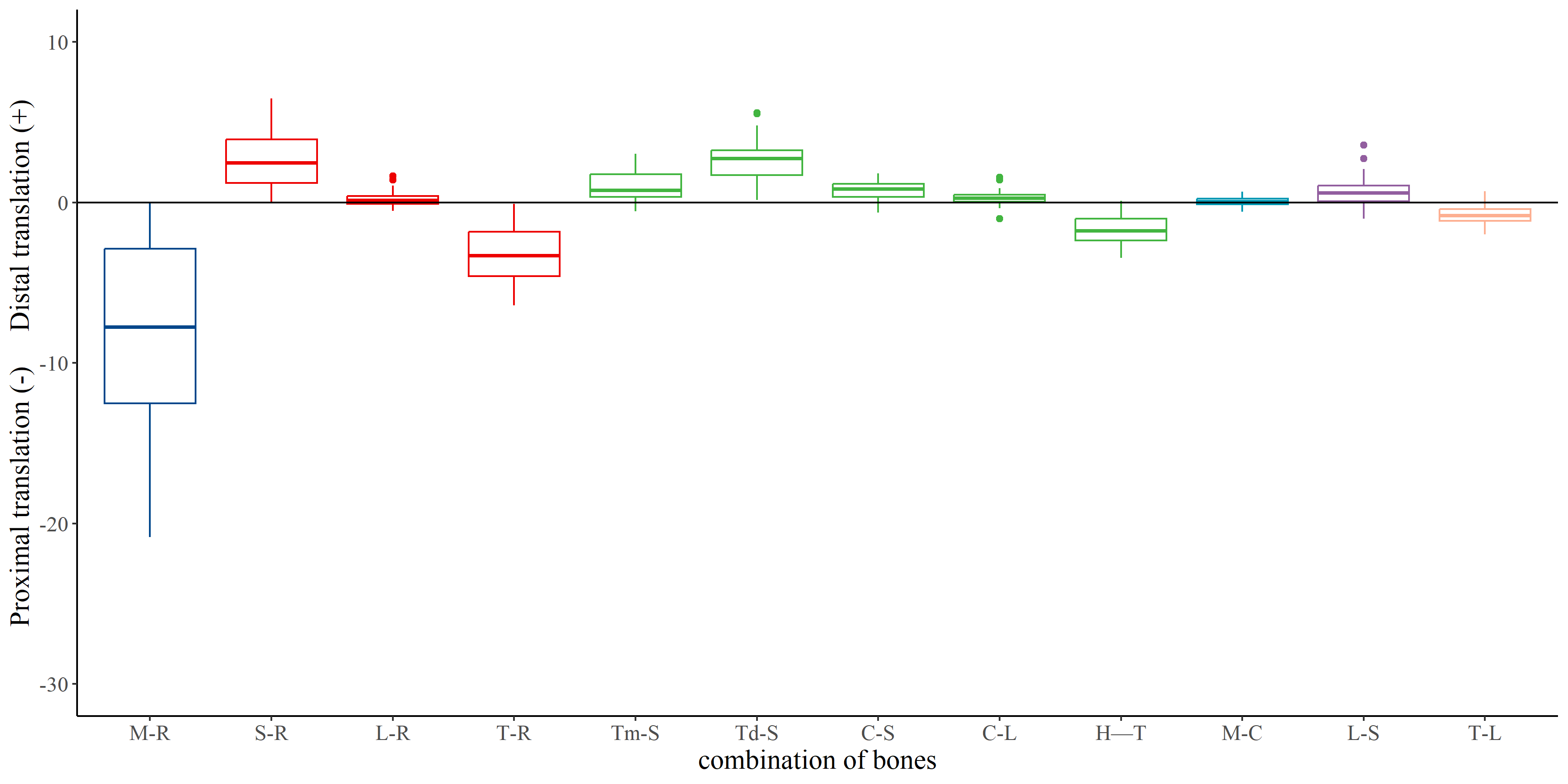


Figure 11. Distal/proximal translations of the twelve analyzed joints during wrist ulnar extension.


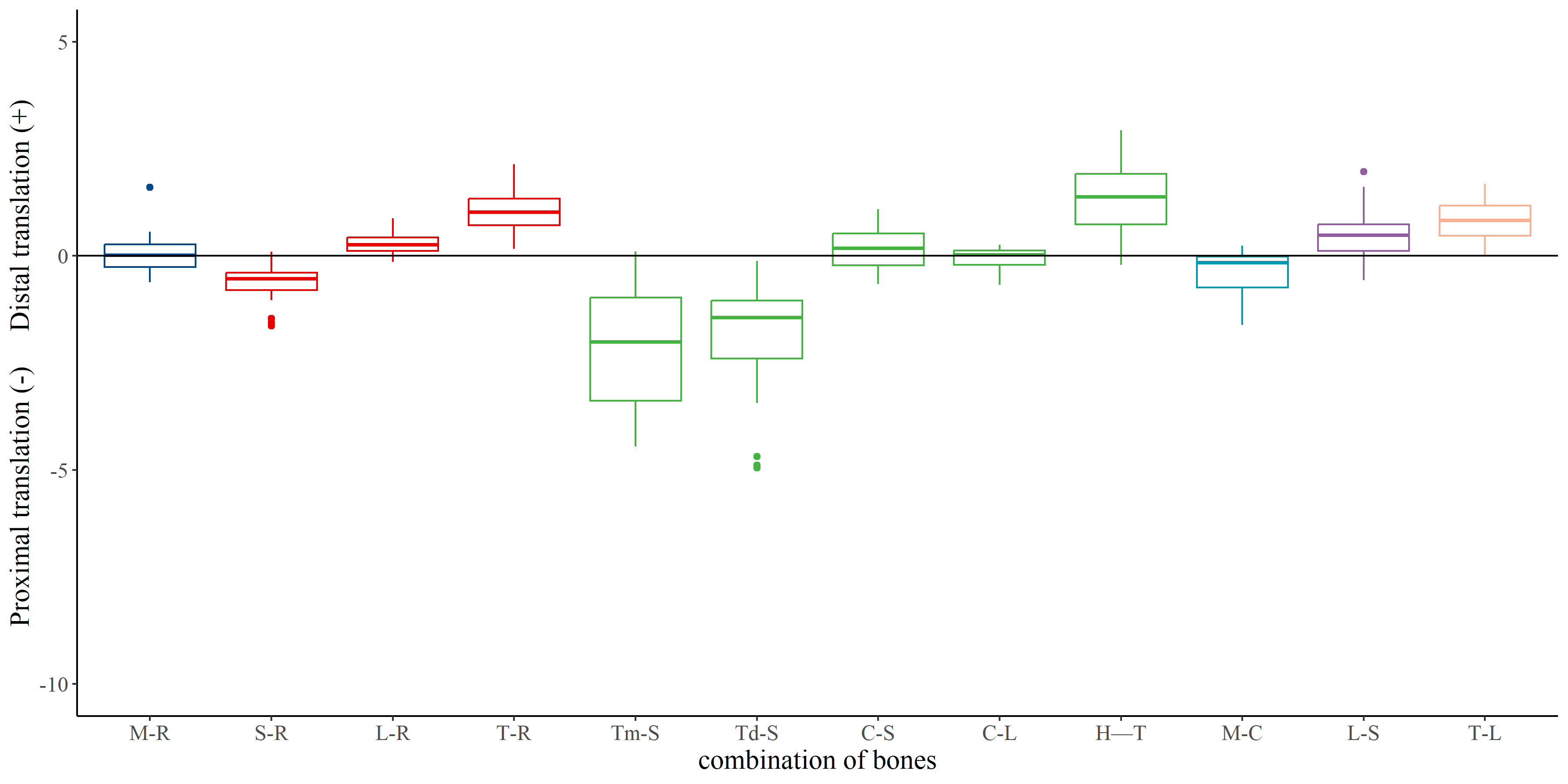


Figure 12. Distal/proximal translations of the twelve analyzed joints during wrist radial flexion.


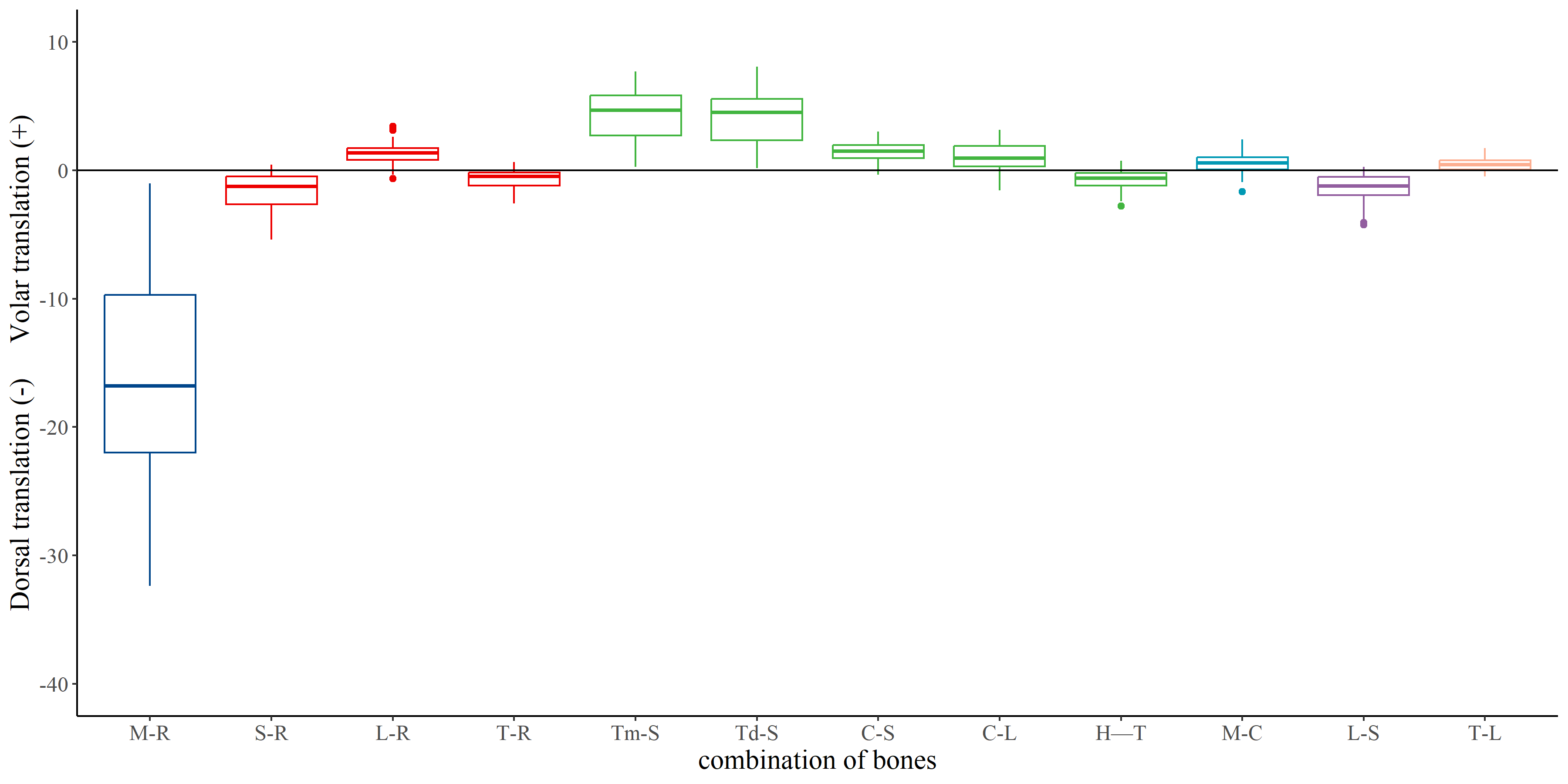


Figure 13. Volar/dorsal translations of the twelve analyzed joints during wrist ulnar extension.


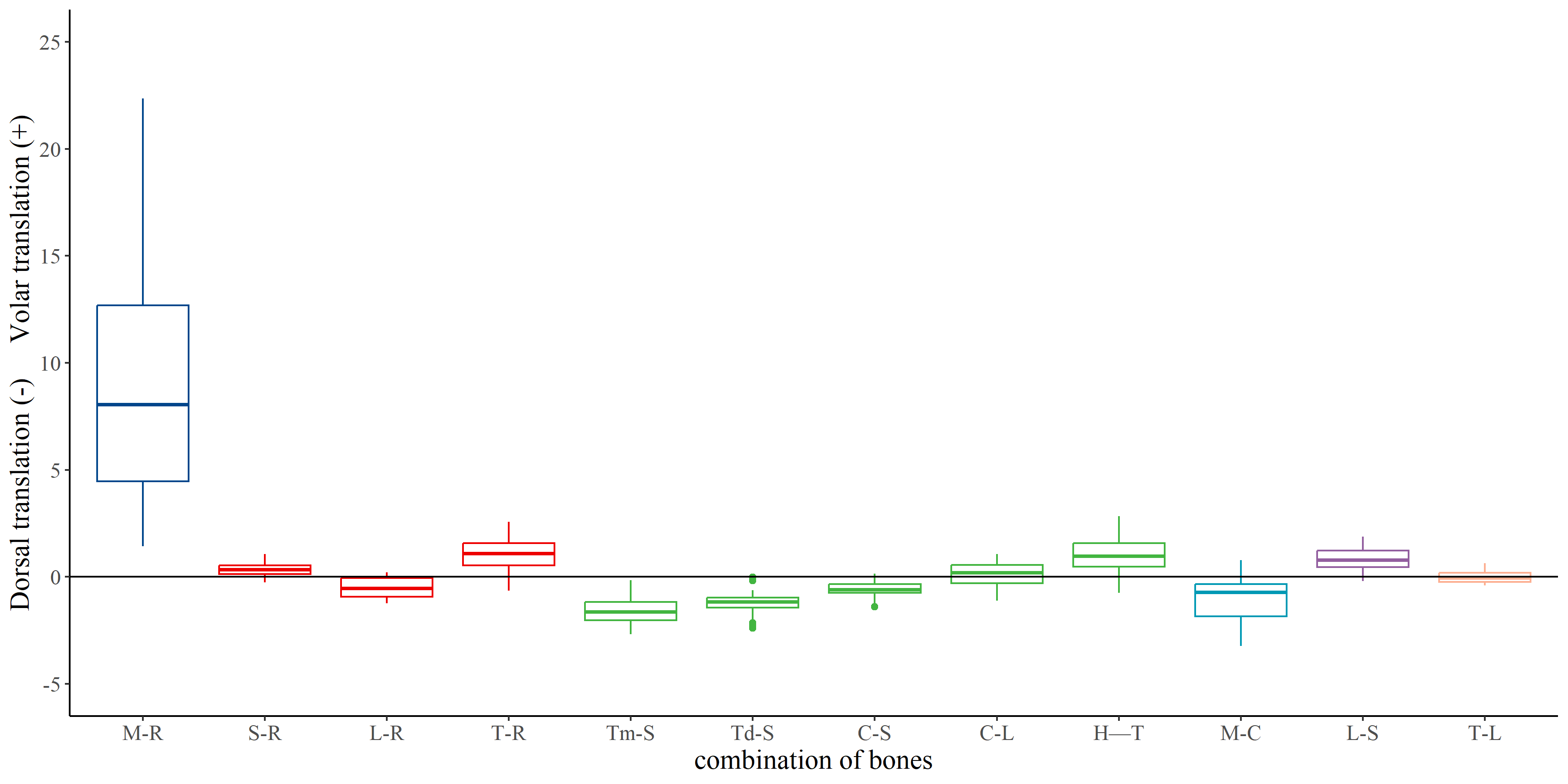


Figure 14. Volar/dorsal translations of the twelve analyzed joints during wrist radial flexion.

Table 1. The p values of comparison of the distal/proximal translations of the twelve joints during wrist ulnar extension.

|  | M-R | S-R | L-R | T-R | Tm-S | Td-S | C-S | C-L | H-L | M-C | L-S |
| --- | --- | --- | --- | --- | --- | --- | --- | --- | --- | --- | --- |
| S-R | <0.001 | NA | NA | NA | NA | NA | NA | NA | NA | NA | NA |
| L-R | <0.001 | <0.001 | NA | NA | NA | NA | NA | NA | NA | NA | NA |
| T-R | <0.001 | <0.001 | <0.001 | NA | NA | NA | NA | NA | NA | NA | NA |
| Tm-S | <0.001 | <0.001 | <0.001 | <0.001 | NA | NA | NA | NA | NA | NA | NA |
| Td-S | <0.001 | 1.000 | <0.001 | 0.001 | <0.001 | NA | NA | NA | NA | NA | NA |
| C-S | <0.001 | <0.001 | <0.001 | <0.001 | 1.000 | <0.001 | NA | NA | NA | NA | NA |
| C-L | <0.001 | <0.001 | 1.000 | <0.001 | <0.001 | <0.001 | <0.001 | NA | NA | NA | NA |
| H-L | <0.001 | <0.001 | <0.001 | <0.001 | <0.001 | <0.001 | <0.001 | <0.001 | NA | NA | NA |
| M-C | <0.001 | <0.001 | 0.114 | <0.001 | <0.001 | <0.001 | <0.001 | <0.001 | <0.001 | NA | NA |
| L-S | <0.001 | <0.001 | <0.001 | <0.001 | 0.792 | <0.001 | 1.000 | <0.001 | <0.001 | <0.001 | NA |
| T-L | <0.001 | <0.001 | <0.001 | <0.001 | 0.522 | <0.001 | 1.000 | <0.001 | <0.001 | <0.001 | 1.000 |

NA: not applicable.

Table 2. The p values of comparison of the distal/proximal translations of the twelve joints during wrist radial flexion.

|  | M-R | S-R | L-R | T-R | Tm-S | Td-S | C-S | C-L | H-L | M-C | L-S |
| --- | --- | --- | --- | --- | --- | --- | --- | --- | --- | --- | --- |
| S-R | 0.064 | NA | NA | NA | NA | NA | NA | NA | NA | NA | NA |
| L-R | 1.000 | 0.021 | NA | NA | NA | NA | NA | NA | NA | NA | NA |
| T-R | <0.001 | <0.001 | <0.001 | NA | NA | NA | NA | NA | NA | NA | NA |
| Tm-S | <0.001 | <0.001 | <0.001 | <0.001 | NA | NA | NA | NA | NA | NA | NA |
| Td-S | <0.001 | <0.001 | <0.001 | <0.001 | 1.000 | NA | NA | NA | NA | NA | NA |
| C-S | 1.000 | 1.000 | 1.000 | <0.001 | <0.001 | <0.001 | NA | NA | NA | NA | NA |
| C-L | 1.000 | <0.001 | 1.000 | <0.001 | <0.001 | <0.001 | 0.097 | NA | NA | NA | NA |
| H-L | <0.001 | <0.001 | <0.001 | 0.018 | 0.002 | 0.019 | <0.001 | <0.001 | NA | NA | NA |
| M-C | 1.000 | 0.269 | 1.000 | <0.001 | <0.001 | <0.001 | 1.000 | 1.000 | <0.001 | NA | NA |
| L-S | 0.238 | 1.000 | 0.008 | 0.001 | <0.001 | <0.001 | 1.000 | 0.016 | <0.001 | 0.321 | NA |
| T-L | 0.001 | 0.599 | 0.001 | 0.060 | <0.001 | <0.001 | 0.002 | <0.001 | <0.001 | 0.033 | 1.000 |

NA: not applicable.

Table 3. The p values of comparison of the extension/flexion of the twelve joints during wrist ulnar extension.

|  | M-R | S-R | L-R | T-R | Tm-S | Td-S | C-S | C-L | H-L | M-C | L-S |
| --- | --- | --- | --- | --- | --- | --- | --- | --- | --- | --- | --- |
| S-R | <0.001 | NA | NA | NA | NA | NA | NA | NA | NA | NA | NA |
| L-R | <0.001 | <0.001 | NA | NA | NA | NA | NA | NA | NA | NA | NA |
| T-R | <0.001 | <0.001 | 0.080 | NA | NA | NA | NA | NA | NA | NA | NA |
| Tm-S | 0.030 | <0.001 | <0.001 | <0.001 | NA | NA | NA | NA | NA | NA | NA |
| Td-S | <0.001 | <0.001 | <0.001 | <0.001 | 0.008 | NA | NA | NA | NA | NA | NA |
| C-S | <0.001 | <0.001 | <0.001 | <0.001 | <0.001 | 0.002 | NA | NA | NA | NA | NA |
| C-L | <0.001 | <0.001 | <0.001 | <0.001 | <0.001 | <0.001 | <0.001 | NA | NA | NA | NA |
| H-L | <0.001 | <0.001 | <0.001 | <0.001 | <0.001 | <0.001 | <0.001 | <0.001 | NA | NA | NA |
| M-C | <0.001 | <0.001 | <0.001 | <0.001 | <0.001 | <0.001 | <0.001 | <0.001 | <0.001 | NA | NA |
| L-S | <0.001 | <0.001 | <0.001 | <0.001 | <0.001 | <0.001 | <0.001 | 1.000 | 1.000 | <0.001 | NA |
| T-L | <0.001 | <0.001 | <0.001 | <0.001 | <0.001 | <0.001 | <0.001 | <0.001 | 1.000 | <0.001 | 0.269 |

NA: not applicable.

Table 4. The p values of comparison of the supination/pronation of the twelve joints during wrist ulnar extension.

|  | M-R | S-R | L-R | T-R | Tm-S | Td-S | C-S | C-L | H-L | M-C | L-S |
| --- | --- | --- | --- | --- | --- | --- | --- | --- | --- | --- | --- |
| S-R | 0.497 | NA | NA | NA | NA | NA | NA | NA | NA | NA | NA |
| L-R | 1.000 | 0.103 | NA | NA | NA | NA | NA | NA | NA | NA | NA |
| T-R | 0.032 | 0.764 | <0.001 | NA | NA | NA | NA | NA | NA | NA | NA |
| Tm-S | <0.001 | <0.001 | <0.001 | <0.001 | NA | NA | NA | NA | NA | NA | NA |
| Td-S | <0.001 | <0.001 | <0.001 | <0.001 | <0.001 | NA | NA | NA | NA | NA | NA |
| C-S | <0.001 | <0.001 | <0.001 | <0.001 | <0.001 | 0.020 | NA | NA | NA | NA | NA |
| C-L | <0.001 | <0.001 | <0.001 | 0.006 | <0.001 | <0.001 | <0.001 | NA | NA | NA | NA |
| H-L | <0.001 | <0.001 | <0.001 | <0.001 | <0.001 | 0.774 | 1.000 | 1.000 | NA | NA | NA |
| M-C | <0.001 | <0.001 | 0.002 | <0.001 | <0.001 | <0.001 | <0.001 | <0.001 | <0.001 | NA | NA |
| L-S | <0.001 | <0.001 | <0.001 | <0.001 | <0.001 | <0.001 | <0.001 | <0.001 | <0.001 | 1.000 | NA |
| T-L | 0.184 | 0.013 | 1.000 | <0.001 | <0.001 | <0.001 | <0.001 | <0.001 | <0.001 | 1.000 | <0.001 |

NA: not applicable.

Table 5. The p values of comparison of the ulnar/radial deviation of the twelve joints during wrist radial flexion.

|  | M-R | S-R | L-R | T-R | Tm-S | Td-S | C-S | C-L | H-L | M-C | L-S |
| --- | --- | --- | --- | --- | --- | --- | --- | --- | --- | --- | --- |
| S-R | <0.001 | NA | NA | NA | NA | NA | NA | NA | NA | NA | NA |
| L-R | <0.001 | 1.000 | NA | NA | NA | NA | NA | NA | NA | NA | NA |
| T-R | <0.001 | <0.001 | <0.001 | NA | NA | NA | NA | NA | NA | NA | NA |
| Tm-S | 0.003 | <0.001 | <0.001 | <0.001 | NA | NA | NA | NA | NA | NA | NA |
| Td-S | 0.186 | <0.001 | <0.001 | <0.001 | 0.667 | NA | NA | NA | NA | NA | NA |
| C-S | 1.000 | <0.001 | <0.001 | <0.001 | <0.001 | 1.000 | NA | NA | NA | NA | NA |
| C-L | 1.000 | <0.001 | <0.001 | <0.001 | 1.000 | 1.000 | 1.000 | NA | NA | NA | NA |
| H-L | <0.001 | <0.001 | <0.001 | 1.000 | <0.001 | <0.001 | <0.001 | <0.001 | NA | NA | NA |
| M-C | <0.001 | 0.286 | 0.036 | <0.001 | <0.001 | <0.001 | <0.001 | <0.001 | <0.001 | NA | NA |
| L-S | <0.001 | 0.041 | 1.000 | 0.011 | <0.001 | <0.001 | <0.001 | <0.001 | <0.001 | <0.001 | NA |
| T-L | <0.001 | <0.001 | <0.001 | 1.000 | <0.001 | <0.001 | <0.001 | <0.001 | 1.000 | <0.001 | 0.001 |

NA: not applicable.
